# *Brassica* Species in Phytoextractions: Real Potentials and Challenges

**DOI:** 10.3390/plants10112340

**Published:** 2021-10-29

**Authors:** Tijana Zeremski, Dragana Ranđelović, Ksenija Jakovljević, Ana Marjanović Jeromela, Stanko Milić

**Affiliations:** 1 Institute of Field and Vegetable Crops, Maksima Gorkog 30, 21000 Novi Sad, Serbia; ana.jeromela@ifvcns.ns.ac.rs (A.M.J.); stanko.milic@ifvcns.ns.ac.rs (S.M.); 2 Institute for Technology of Nuclear and Other Mineral Raw Materials, Franchet d’Esperey Boulevard 86, 11000 Belgrade, Serbia; d.randjelovic@itnms.ac.rs; 3 Institute of Botany and Botanical Garden, Faculty of Biology, University of Belgrade, Takovska 43, 11000 Belgrade, Serbia; kjakovljevic@bio.bg.ac.rs

**Keywords:** *Brassicaceae*, biomass, metal, assisted phytoextraction, field trials, contaminated soil

## Abstract

The genus *Brassica* is recognized for including species with phytoaccumulation potential and a large amount of research has been carried out in this area under a variety of conditions, from laboratory experiments to field trials, with spiked or naturally contaminated soils, using one- or multi-element contaminated soil, generating various and sometimes contradictory results with limited practical applications. To date, the actual field potential of *Brassica* species and the feasibility of a complete phytoextraction process have not been fully evaluated. Therefore, the aim of this study was to summarize the results of the experiments that have been performed with a view to analyzing real potentials and limitations. The reduced biomass and low metal mobility in the soil have been addressed by the development of chemically or biologically assisted phytoremediation technologies, the use of soil amendments, and the application of crop management strategies. Certain issues, such as the fate of harvested biomass or the performance of species in multi-metal-contaminated soils, remain to be solved by future research. Potential improvements to current experimental settings include testing species grown to full maturity, using a greater amount of soil in experiments, conducting more trials under real field conditions, developing improved crop management systems, and optimizing solutions for harvested biomass disposal.

## 1. Introduction

Environmental pollution has been emphasized in recent decades as one of the main consequences of rapid development, the generation of large amounts of waste containing high levels of contaminants, such as metal/oids, pesticides, radionuclides, polyaromatic hydrocarbons, various types of leachates, etc., being one of the major sources [1,2]. As the quantities of contaminants increase over time, their removal becomes more and more important. Although waste disposal without any treatment to reduce pollution is the simplest solution—especially when it comes to mining activities, which is one of the largest anthropogenic sources of metals and metalloids—the potential toxicity of waste makes its remediation imperative [3]. The use of plants in reducing pollution, i.e., phytoremediation, is the most acceptable method of decontamination from an environmental point of view. In this process, plant species are used to remove pollutants or render them harmless by extraction, sequestration, degradation, or detoxication [4,5,6]. Depending on how pollutants are removed, several techniques of phytoremediation can be identified: phytoextraction, phytostabilization, phytovolatilization, phytofiltration, and phytodegradation [7,8]. Phytoextraction has proven to be the most useful and efficient in the process of removing metals from contaminated sites [9]. Phytoextraction (phytoaccumulation) is the process of removing pollutants through their intensive uptake by the roots and their accumulation in the aboveground parts of hyperaccumulating plants [10]. For successful application in the phytoextraction process, a species must be a (hyper)accumulator of a certain element to efficiently extract metal/oids from the soil and transport them to the shoot [11]. In addition to the preference for certain elements, it is advantageous if the plant has an extensive root system and a large biomass in order to access and accumulate as many elements as possible. Species with a large biomass can be used efficiently in phytoextraction even if they do not have hyperaccumulation potential, especially if they are deep-rooted and fast-growing [12].

A considerable number of hyperaccumulators belong to the *Brassicaceae* and they have been widely used in the phytoremediation process [11,13,14]. This family encompasses more than 330 genera and 3700 taxa, distributed all around the world, and is of great economic importance [15,16]. Although the representatives of this family are known mainly as crops and for their use in human nutrition, they are also used in medicine and as ornamental plants [16]. The accumulation of heavy metals is particularly common to members of the family, including Cd, Pb, Zn, Se, Ni, etc. [17]. Of the 721 taxa found to date to be able to hyperaccumulate one or more metal/oids in their aboveground tissues, more than 100 belong to the family Brassicaceae [18,19]. Most of these species hyperaccumulate Ni and Zn (72 and 20, respectively) and the largest number of hyperaccumulators was found within the genera *Noccaea* and *Odontarrhena* [15]. Within the genus *Odontarrhena*, a total of 62 hyperaccumulator species have been recorded, of which 48 hyperaccumulated Ni [20]. More importantly, species of the genus *Noccaea* show the ability to accumulate multiple elements, a trait that rarely occurs in nature [21]; however, due to restricted growth of the root system and low biomass, their use in phytoremediation is not cost effective [22,23].

At the same time, there are non-accumulator Brassicaceae species that can also tolerate high concentrations of heavy metals in their shoots. For this purpose, the species of the genus *Brassica*, have been used particularly intensively. This genus comprises 39 recognized species [24] distributed mainly in the Mediterranean region, in most parts of Europe (Central, Western, and Eastern Europe), and in Central and Eastern Asia [25]. According to Ball et al. [26], a total of 22 species of the genus *Brassica* occurs in Europe, as native, cultivated, or even as a weed, of which 10 have been recorded on the Balkan Peninsula. Most of these species are mainly used for human and animal nutrition. While the vegetative parts are mainly used as a raw product, generative ones are mainly processed into oil, spices, flour, protein, etc. [27]. A number of *Brassica* species show a certain potential for metal/oid accumulation, including Cg, Pb, Zn, Cu, Ni, etc. [28]. Although not so efficient in accumulation, due to their large biomass and rapid growth rate, these species are able to extract a sufficient amount of metal to be successfully used in the phytoremediation process. Species such as *Brassica juncea* (L.) Czern., *Brassica carinata* A. Braun, *Brassica napus* L., *Brassica nigra* (L.) K. Koch, *Brassica rapa* L., and *Brassica oleracea* L. have been particularly intensively researched [28,29,30,31,32]. *Brassica juncea* (Indian mustard) is among the most frequently investigated *Brassica* species in terms of potential applications in phytoremediation processes [17]. This species formed by crossing *Brassica nigra* and *Brassica rapa* is predominantly grown in arid and semi-arid regions and mainly used for oilseed production [16]. At the same time, it efficiently accumulates heavy metals (Ni, Zn, Pb, and Cd) which are translocated to the shoots [33]. The significant phytoremediation potential was also observed in *Brassica napus* (rapeseed), a species widely used as a source of edible oil and biodiesel due to the high content of oil (>40%) in its seed [34]. By combining these two processes, the cost-effectiveness of *Brassica napus* usage greatly increases. This species is thought to originate from China and Central Asia, similar to *Brassica nigra* (black mustard). However, since it is considered a noxious weed, domestication of *Brassica nigra* has been rather limited [16]. *Brassica rapa* (syn. *Brassica campestris* L.) is a mountainous sub-Mediterranean species of significant agricultural importance, cultivated worldwide as an oilseed crop [35]. There are several different varieties within this species which are sometimes considered to be distinct species. Some of these are *Brassica rapa* var. *pekinensis* (Chinese cabbage), *Brassica rapa* var. *chinensis*, *Brassica rapa* var. *japonica*, and *Brassica rapa* var. *rapa*. *Brassica oleracea* is of similar origin (i.e., Mediterranean) and it is native to the coastal area of the Mediterranean Sea and Atlantic Ocean. As a cultivated form, it is distributed all over the world, except in the tropics [36]. Some of the varieties of this species that have been studied are *Brassica oleracea* var. *capitata* f. *alba* [37] and *Brassica oleracea* var. *acephala* [38]. By crossing with *Brassica nigra*, amphidiploid *Brassica carinata* (Ethiopian mustard) is formed. The center of diversity of this species is in Ethiopia, where its cultivation started [39]. It adapted well to dry areas, requiring significantly less precipitation than other *Brassica* species, and can survive in nutrient-poor and highly saline conditions [40,41].

Although *Brassica* species have certain characteristics which make them candidates for use in phytoextractions, there are a number of factors that limit the efficiency of the phytoextractive processes in which they are used. The aim of this review was to summarize the results of experiments on *Brassica* species used for the phytoextraction of metal contaminated soils and to analyze their potential and limitations.

## 2. Basis of Tolerance to Heavy Metals in *Brassica* Species

Representatives of the Brassicaceae family show different tolerance strategies to elevated heavy metal concentrations, from excluders to accumulator and hyperaccumulator species [42]. The tolerance of plants to elevated concentrations of heavy metals in the substrate is underpinned by two basic mechanisms. One is the avoidance of metal uptake or the chelation and sequestration of metals in the vacuoles; the other involves the activation of antioxidant mechanisms [43]. The exclusion strategy involves limiting heavy metal uptake by root exudates secreted actively or passively, whereby such compounds bind metal ions to the surface of roots and prevent their adoption [44]. Additionally, an important detoxification mechanism of many plants is based on chelation, a process in which phytochelatins (PCs) and metallothioneins (MTs) play important roles as ligands, including amino acids, organic acids, and various phosphate derivatives which bind metal ions and sequester them in vacuoles. Analyses by Nazir et al. [44] have shown that the strongest induction of PCs is caused by Cd. A complex of Cd and PCs is formed via the thiol group of cysteine, the compartmentalization of which in the vacuoles reduces the amount of Cd that can be freely transported through the plant. The induction of PCs is also caused by other elements, such as As, Cu, Zn, Ni, Cr, Hg, and Se, and the existence of complexes of these ions with PCs have been demonstrated as detoxification mechanisms in several species of the genus *Brassica*, e.g., *B*. *napus* for Cd and Se [45,46], *B*. *chinensis* for Hg [47], and *B*. *juncea* for As [48]. Reduced glutathione (GSH) is recognized as a significant component in the synthesis of PCs, and it has an important role in the tolerance to elevated heavy metal concentrations of various plant species [49]. For example, the addition of sulfur caused a reduction of Cd concentration in edible parts of *B*. *chinensis*, leading to increased expression of the GHS1 gene and thus to an increase in the amount of GSH and greater chelation of Cd, which was then sequestered in vacuoles [50]. Sequestration as a survival mechanism on metal-rich substrates was observed in the same species under elevated Pb concentration, where accumulation of Pb was predominantly found in vacuoles of the root, while lower Pb concentration were deposited in vacuoles in the shoot [51]. The same strategy was observed in *B*. *juncea* after irrigation with river water contaminated with Hg, Cd, Pb, Ni, and Zn [52]. Sequestration has been confirmed as an important mechanism for tolerance of elevated heavy metal concentrations in *B*. *juncea* growing on a Cd-rich medium. As overcoming stress caused by heavy metals is an energy-consuming process, depletion of growth was observed in *B*. *juncea*. Changes in photosynthetic activity contributed to growth reduction as chlorophyll and carotenoid content decreased [53]. Stress conditions, such as the presence of heavy metals at elevated concentrations, initially induce a response in plants in the form of an excessive production of reactive oxygen species which causes an oxidative response [54]. The consequences of oxidative stress are manifested in an alteration of enzyme function (dysfunction or inactivation), oxidation of proteins, lipid peroxidation of cell membranes, and damage to the plant tissue structures [44]. The damage caused by active oxygen is removed by various antioxidant mechanisms, such as peroxidase (POD), superoxide dismutase (SOD), glutathione peroxidase (GPX), catalase (CAT), phenolic compounds, alkaloids, etc., which increases the tolerance of *Brassica* species to elevated concentrations of various metals [55]. Moreover, altered tolerance to Cr, Fe, Mn, and Zn in *B*. *juncea* cv. Rohini exposed to tannery sludge resulted from the overproduction of antioxidants as increased concentrations of proline, ascorbic acid, cysteine, and malondialdehyde were observed in the root and leaves [55]. Moreover, the degree of tolerance has been shown to vary not only among species of the genus *Brassica*, but also among different genotypes of the same species, as different genotypes of *B*. *juncea* showed various responses to Al-induced oxidative stress, presented by different amounts of proline as well as by the strength of the non-enzymatic and enzymatic antioxidant defense system [56]. In order to determine the biochemical basis of HM tolerance in species of the genus *Brassica*, it is important to highlight the individual amino acids that function as ligands in metal detoxification and thus also in plant tolerance [57]. In this respect, cysteine and histidine are particularly important as their concentration was found to increase in the presence of elevated Ni concentrations. A similar relationship between cysteine and Ni was demonstrated by [58]. The association of amino acid and Ni concentrations has also been demonstrated in other species of the Brassicaceae family [59,60].

The predominant strategy of plants living in hostile environments rich in heavy metals is exclusion as a mechanism to avoid potential damage that would occur after the adoption of metals, especially in photosynthetic organs [61]. In rare cases, plant species tolerant to excessive concentrations of heavy metals in the soil may absorb them in aboveground tissues at concentrations above the notional threshold, representing hyperaccumulators of certain elements. Genes responsible for the uptake and transport of metals, the synthesis of chelators that bind metals which are then sequestered into shoot vacuoles, and the strength of the oxidative stress response were found to be expressed more in hyperaccumulating plants then in less metal-tolerant species [61]. These findings suggest that hyperaccumulation represents an adaptation based on a change in metal homeostasis [62]. Considering that the highest number of hyperaccumulator plant species have been discovered within the Brassicaceae, research on the diverse processes underlying hyperaccumulation are specifically extensive within this family [63,64]. A particularly common phenomenon among *Brassica* species is the hyperaccumulation of Se. A total of 1200 μg/g Se has been observed in flowers of *B*. *oleracea* [65], whereby selenocysteine methyl transferase has been shown to be responsible for hyperaccumulation under conditions of soil Se excess [66]. In *B*. *juncea*, recognized as hyperaccumulator of Cd, tolerance to a high content of this element is related to changes in photorespiration. It has been found that an increase in photorespiration rate hinders photoinhibition, caused by Cd-induced decrease in stomatal conductance [67].

## 3. Assessing *Brassica* Species Potential for Phytoextraction through Experiments with Spiked vs. Naturally Contaminated Soils

Studies of the uptake of metals in plants during the phytoremediation process are often made using uncontaminated soils spiked with known concentrations of selected metals rather than aged and contaminated field soils. However, there are uncertainties about how well the distribution of metals in artificially spiked soils resemble the distribution in field soils, and this is a factor which can have a significant influence on the final results [28,68]. Although metals originating from anthropogenic sources tend to be in more mobile forms in field soils than those of lithogenic origin, metals in artificially spiked soils are usually present in reactive forms [69]. For instance, the uptake of Cu from freshly spiked and aged soil in *B. juncea* was assessed for phytoremediation purposes [70]. Plant biomass had significantly decreased (>50%) in freshly spiked soil and the amount of Cu uptake was higher (approximately 15%) in comparison to the aged soil, showing that removal rates obtained from experiments with freshly spiked soils may differ from the removal rates from soils gained in real field conditions.

Metal bioavailability is thought to be influenced by various soil characteristics, as well as the aging period. The role of organic matter, pH, Fe/Mn hydroxides, and clay minerals is well recognized by different prediction models [71]. However, broader research and toxicity assessments suggest that living components of the pedosphere, including microorganisms, plants, and soil invertebrates, also play a significant role in metal stabilization, revealing the role of biotic factors in metal-contaminated soils [71,72]. Aging processes tends to stabilize metals in soils by decreasing their availability with time [73]. For instance, Lu et al. [74] showed that aging significantly affected the distribution of Cu among the fractions of artificially spiked soil such that it was incorporated in more stable fractions, while it had very little effect on Cu bioavailability to *Triticum aestivum* and *Eisenia fetida* when they were used as test organisms. The results of this research suggest that biological control may be more important for the bioaccumulation of Cu in comparison to the changes in Cu fractions caused by soil aging. It is known that diverse microorganisms can influence metal availability and uptake, allowing them to be used for bioremediation and enhanced phytoremediation purposes [75,76]. However, Moreira et al. [77] found that the selection of plant growth promoting rhizobacteria (PGPR) for phytoremediation purposes on spiked and field-contaminated sterilized soils showed significant differences, so that if assessed only in spiked and sterilized soils, the effect of inoculants may be overestimated and not be suitable for application in real field conditions.

Even if contaminated field soil is used in the experiments, the experimental setting may also significantly contribute to the results obtained. Greenhouse pot and field studies conducted to optimize Cr, Cu, Pb, and Zn phytoextraction by *B. napus*, coupled with the addition of *Bacillus licheniformis* and the addition of municipal solid waste, showed that *B. napus* posed different accumulation patterns under different growing conditions [78]. In a pot experiment, the accumulation of metals was higher in the shoots than in the roots of the plant exhibiting phytoextraction characteristics (with TF > 1, up to 5.04 in the case of Pb), whilst in the field conditions the roots accumulated more metals than the shoots (with TF < 1 in almost all treatments and elements on contaminated sites, excluding Cd with TF = 1.17), where *B. napus* showed phytostabilization potential.

## 4. The Efficiency of *Brassica* Species for Phytoextraction of Multielement Contaminated Soils

Industrial development followed by an increasing rate of anthropogenic activities, such as mining, smelting, the use of chemicals in agriculture, the burning of fossil fuels, vehicle exhaust emissions, etc., has caused diverse levels of soil contamination, often by multiple pollutants of organic or/and inorganic origin. Various research has been conducted to assess the phytoextraction efficacy of *Brassica* species, on both natural, multi-contaminated soils and on artificially spiked soils in laboratory or greenhouse conditions [79,80,81]. Multi-contaminated soils present complex environments for studying phytoremediation effects. It is not only diverse soil variables that affect element uptake (soil type, pH, presence of organic matter and ligands, type of clay, etc.), but also the mutual interactions between soil elements (synergistic, additive, or antagonistic) can contribute significantly to element mobility, uptake and transport in plants [82,83]. For example, synergistic effects of Cu and Cr on metal uptake, oxidative stress, and antioxidant responses in *B. napus* cultivars were revealed by Li et al. [84]. However, certain metals may have similar affinities for binding sites in plant tissues showing similarities in accumulation patterns, while under higher concentrations their behavior may become antagonistic [85]. Due to such constraints, there has been less research describing phytoaccumulation patterns on multi-metal-contaminated soils in natural conditions compared with single element contamination in controlled settings, although previous reports have represented the actual case of land remediation [86].

Several *Brassica* species are recognized for their potential to accumulate metals, and certain phytoextraction studies on mono-metal soil contamination showed promising results, such as an increase in metal content in plant roots or a transfer factor from root to shoot following elevated concentrations of metals in soil [87,88]. However, the behavior of *Brassica* species under conditions of multi-metal-contaminated soils has been assessed to a far lesser extent [89]. The phytoremediation potential of six different plant species (including *B. juncea*) in soils contaminated with Cd, Cr, Ni, and Zn showed that metal accumulation was specific to each plant species [90]. Moreover, not only did *B. juncea* accumulate more Cd, Cr, Ni, and Zn in the shoots than in the roots, but its accumulated concentration of Ni in both roots and shoots (1133 mg/kg and 2784 mg/kg, respectively) was several-fold higher than in other investigated species, crossing the hyperaccumulation threshold values for Ni. In addition to a revealed potential for phytoextraction of Ni, Zn, and Cd (exhibiting BCFs of 4.46, 2.21, and 1.75 and TFs of 2.46, 2.45, and 4.5, respectively), the results showed that *B. juncea* is able to compensate lower remediation effectiveness for certain elements by producing a 1.2–4.8 times higher amount of biomass (22.5 g per plant on average) than some of the investigated species belonging to the other genera. Contrary to this, the investigations of Marchiol et al. [86] on phytoextraction of Cd, Cr, Cu, Ni, Pb, and Zn multi-contaminated soils with *B. napus* showed that this species has limited phytoextraction potential for sites exhibiting TF < 1 for all the investigated elements, reducing its biomass by up to 47% in comparison to the control. Gisbert et al. [91] pointed out similar differences among *Brassica* species and their cultivars based on a tolerance index expressed as shoot fresh weight production rate: *B. juncea* (~98%) > *B. carinata* cultivar 117 (~74%) > *B. carinata* cultivar 2920 ≈ *B. oleracea* (~66%). Assessment of different genotypes of *B. juncea* in phytoremediation of Cd and Pb contaminated field soils revealed statistically significant differences among 80 cultivars for Cd and Pb uptake [14]. Generally, the average uptake of Cd and Pb was higher in shoots than in roots, exhibiting TF values for Cd in the range of 0.22–3.38 and for Pb in the range of 0.48–3.87, showing a potential for phytoremediation in low to moderate multi-contaminated sites. Moreover, a significant positive correlation between produced shoot biomass and total Cs and Pb uptake in *B. juncea* cultivars was observed in this study (r = 0.7 and r = 0. 66, respectively, at a confidence level of *p* < 0.01), confirming that aboveground biomass production was one of the factors that influenced diversified results for metal accumulation among the tested genotypes. Similar findings were made by Podar et al. [92], who tested the effects of heterogeneity in Cd and Zn polluted soils on metal uptake by *B. juncea*. The results showed that a heterogeneous distribution of Zn in Cd-contaminated soils resulted in selective placement of the plant root system, causing a 1.6- to 24-fold increase in shoot biomass and consequently its total metal content (4 to 10 times higher than in Cd contaminated soils with homogenous distribution of Zn).

Many researchers stipulate that certain *Brassica* species should only be used in cases of low to moderate multi-element contaminated soils, due to limited extraction or biomass production in the case of the simultaneously increased content of several elements [14,28,89]. Additional investigation of promising *Brassica* species in diverse conditions of multi-element contaminated soils could point to the specific site assets and the species that can combine an increase in biomass production and phytoextraction capabilities for several metals.

## 5. *Brassica* Species and Enhanced Phytoextraction

In order to enhance the phytoremediation potential of *Brassica* species, research has progressed in several directions, including the development of practices such as crop management patterns and chemically or biologically assisted remediation.

### 5.1. The Use of PGPR

Plant growth promoting rhizobacteria (PGPR) were primarily used in agriculture to increase the productivity and to protect plants against stress caused by drought, floods, high salinity, phytopathogens, etc. [93]. PGPR suitable for enhancing heavy metal phytoextraction need to be tolerant to high concentrations of heavy metals in the soil. This tolerance is achieved by developing mechanisms to reduce the toxicity of metal ions by transforming them into less toxic forms or by metal sequestration in extracellular or intracellular polymers [94]. Metal tolerant PGPR influence plants by producing plant hormones (gibberellins, cytokinins, auxins), through the secretion of siderophores which can alter nutrient and metals bioavailability, but the most important effect of PGPR is an increase of plant tolerance to high heavy metal concentrations in the soil through the regulation of ethylene concentrations through the synthesis of ACC deaminase [95]. Plant growth promoting endophytes (PGPE) are microorganisms that colonized in plants and are beneficial for their growth and hardiness [96].

PGPR used in phytoextraction experiments are usually isolated from the rizosphere of plants growing in polluted soils [97,98,99,100,101] and are thus adapted to high concentrations of metals, while PGPE used for enhancing phytoextraction are usually isolated from hyperaccumulator plants or other plants growing in polluted soils [102,103]. Recent examples of PGPR/PGPE applications in phytoremediation with *Brassica* species are presented in Table 1.

The promotion of root and shoot growth is one of the main effects of PGPR on plants. Various studies report increases in plant total biomass from 20 to 60% after PGPR application [106,107]. However, this effect was not observed in some recent studies on PGPR-enhanced phytoextractions with *Brassica* species [98,100,104,105]. Jinal et al. [101] found that inoculation of *Brassica juncea* seeds with iron-tolerant PGP bacteria enhanced the root length of plants grown in iron contaminated soil from 47.1 to 106.4% and shoot length from 49.40 to 71.71% compared to controls. A phytoremediation study conducted on multi-metal-contaminated mine tailings soil using *B. juncea* inoculated with five different plant-growth promoting bacteria strains showed that inoculation of plants increased stem height by 1.5 to 2.0 times and root growth by 2.7 up to 5.2 times compared to controls [98]. Increased biomass of *B. napus* grown in soil polluted with Cd and Pb was observed after inoculating *Brassica* seeds with the endophytic fungi *Fusarium* sp. CBRF44 and *Penicillium* sp. CBRF65 from the roots and stems of *B. napus* grown in contaminated soils [103]. Similarly, in a study on the effect of inoculation of *Brassica napus* with a PGPE consortium on the phytoextraction of Cd from cadmium polluted soil, an increase of the total biomass of oilseed rape by 11.3, 10.2, and 20.0%, respectively, was observed in three cropping years [102].

The effect of PGPR and PGPE on enhanced uptake of heavy metals by *Brassica* species was reported in most of the recently published studies [99,100,101,102,103,104,105,106], but the efficiency of uptake varied depending on the metal. A positive effect of PGPR inoculation on the uptake and translocation of Cd and Zn and no effect on the uptake of Cu and Pb in *B. juncea* was observed in an experiment with bacterial strains belonging to the genera *Burkholderia* containing the enzyme ACC deaminase which controls the production of ethylene in plants and might cause an enhanced uptake of metals through increasing tolerance of stress by reducing ethylene concentration in plants and through modification of root architecture [104]. Similarly, *B. napus* inoculated with *Bacteroidetes bacterium*, *Pseudomonas fluorescens* and *Variovorax* sp. and grown in soil contaminated with Cd, Zn, Pb, and Cu, showed an increased uptake of only Cd and Zn. Mendoza-Hernández et al. [97] used different PGPR isolated from the rizosphere of plants grown in heavily contaminated mine tailing for inoculation of *B. juncea* plants and grown in multielement contaminated mine tailings. Inoculation with *Serratia* K120 resulted in the highest concentrations of Al, Fe, Pb, Cd, Cu, Cr, Mn, and As in roots, and additionally favored the transfer of all elements to the plant aerial part in comparison to other used strains, while inoculation with *Enterobacter* MC156 promoted phytostabilization of selected elements in plant roots.

### 5.2. Chemically Assisted Phytoextraction

The low bioavailability of heavy metals in contaminated soils is one of the biggest limitations on phytoextraction. One of the most frequently explored strategies to increase the efficiency of phytoextraction is using chemical compounds to increase metal bioavailability in soil. These chemical compounds are called chelating agents or chelators and their most important characteristic is the ability to form several bonds with a single metal, thus forming a stable metal–chelator complex that is soluble in the soil solution and thus bioavailable to plants.

Several different chelator types have been investigated for applications in enhanced phytoextractions, but the most efficient and the most frequently studied are chelators belonging to the aminopolycarboxylic acids group (APCA), represented by EDTA (ethylene diamine tetraacetic acid) and EDDS ([S,S]-isomer of ethylenediamine disuccinate), and chelators belonging to the natural low molecular weight organic acid group (NLMWOA), represented by gluconic (GA), oxalic (OA), malic (MA), succinic (SA), citric acid (CA), and N,N-Bis(carboxymethyl)-L-glutamate acid (GLDA).

The application of chelators increases the overall mobility of heavy metals in soils and thus the risk of their leaching into deeper layers of soil. The risk of heavy metal leaching is particularly pronounced when using EDTA due to its very low biodegradability. The process of EDTA–metal complex biodegradation in soil starts approximately one month after EDTA application [108] and the estimated degradation half-time in soil is 6 months [109]. Of the most commonly used chelators, the highest biodegradability has citric acid with a half-life of 2–6 days and a cumulative degradation of 80% within 14 days [110].

Numerous studies on the effect of applications of different chelating agents on metal uptake and translocation by *Brassica* sp. [89,111,112,113,114,115,116] addressed the pronounced phytotoxicity which affects plant biomass production as one of the most frequently observed side effects of increased metal bioavailability. The influence of the application of different chelators on biomass reduction in cultivated *Brassica* species is presented in Table 2.

Based on data presented in Table 2 it can be concluded that biomass production is strongly influenced by the chelator application rate. Chelators applied in low doses, e.g., 2 mmol/kg EDDS and EDTA [111] and 3 mmol/kg GLDA [89], did not influence plant growth, but the higher concentrations significantly affected biomass production. The highest reduction in biomass (72.6%) was observed in *B. juncea* growing in soil treated with 10 mmol/kg EDTA and *B. napus* growing in soil treated with 8 mmol/kg EDDS.

The ability of the chelator to build a stable complex with a specific metal in the soil is also a very important factor which determines the phytotoxicity of the targeted metal and its influence on biomass production. For example, compared to CA, EDTA has a higher affinity with Pb in the soil since it builds a more stable complex with Pb (the stability constant (logKs) for EDTA–Pb is 18 and for CA–Pb is 4.08) [117]. For this reason, the phytotoxic effect of Pb on biomass production of *B. juncea* was more pronounced after treating Pb contaminated soil with EDTA than with CA [115].

Different *Brassica* species have different defense mechanisms with which to respond to the harmful effect of heavy metal induced stress. Species that are more tolerant to heavy metals suffer less biomass reduction. For example, *B. juncea* and *B. rapa* grown under the same conditions responded to an increased bioavailability of heavy metals with different biomass reductions. The reduction of biomass was higher in *B. juncea* (40%) compared to *B. rapa* (13%) [89].

The efficiency of phytoextraction is defined by several parameters: metal concentration in plant tissues, bioconcentration factor (BCF), and translocation factor (TF). BCF is an index which describes the ratio of heavy metal concentration in plant tissues to the heavy metal concentration in soil and is a measure of the ability of a plant to take up heavy metals from soil. In contrast, TF describes the ability of a plant to translocate heavy metals from the roots to the aboveground parts of plants (shoots, stems, leaves, and flowers). The metal concentrations in shoots, BCFs and TFs reported in various studies on the effects of the application of different chelating agents on metal uptake and translocation by *Brassica* species are summarized in Table 3. Based on the results of the presented studies it can be concluded that increased plant metal uptake was the main effect of chelator application.

The main factors influencing heavy metals uptake by *Brassica* plants are the concentration of the applied chelators and their ability to build stable and soluble complexes with targeted metals in the soil. Increasing the concentration of chelators increases the uptake of metals by plants, but this effect is less pronounced at higher chelator concentrations due to the strong phytotoxic effect of heavy metals. For example, Zeremski et al. [111] reported an insignificant difference in Cu uptake by *B. napus* after soil treatment with EDTA at concentrations of 4 and 8 mmol/kg. A similar phenomenon was observed by Di Guo [112] in Cd and Zn uptake by *B. juncea* after treatment with EDTA at concentrations of 5 and 10 mmol/kg and after soil treatment with a combination of 5 mmol/kg EDTA with 5 and 10 mmol/kg OA.

It has already been mentioned that the affinity of a chelator with a specific metal in the soil is also a very important factor that determines the availability of the metal for plant uptake. EDTA builds stable complexes with the majority of heavy metals, which makes it one of the most efficient chelators, and that is the main reason why much research on the use of EDTA in phytoextractions is conducted even though it has low biodegradability, and its application can pose a risk to the environment. However, EDTA has low selectivity and can sometimes react with metals other than those targeted. For example, in calcareous soils, high concentrations of Ca strongly interfere with the process of heavy metals binding with EDTA. Zeremski et al. [111] studied the efficiency of EDTA and EDDS in enhancing Cu uptake by *B. napus* grown in calcareous soils and found that, although the stability constants of EDTA–Cu and EDDS–Cu were almost the same (18.7 and 18.4, respectively), EDDS application increased Cu concentration in *B. napus* shoots to 316.4 mg/kg, whereas EDTA increased the Cu concentration to only 52 mg/kg. The reason for this significant difference between EDDS and EDTA efficiency in Cu mobilization in calcareous soil lies in the much higher affinity of EDTA with Ca (logK EDTA–Ca is 10.6 and logK EDDS–Ca is 4.2) [118]. For this reason, in soils rich in Ca most of the EDTA is used for building complexes with Ca and the targeted heavy metals are mobilized to a lesser extent.

The influence of chelator application on bioconcentration and translocation factors for different metals in *Brassica* species is presented in Table 3. The application of chelators increased metal uptake by plants which led to an increase of BCF. However, in some experiments, despite the increase, BCF values remained lower than 1 indicating that the concentration of metal in the plant tissues (roots and aboveground parts) remained lower than in the surrounding soil. A similar phenomenon was observed for translocation factor values. A translocation factor higher than 1 indicates that the metal predominantly accumulates in the harvestable parts of the plant, which is of great importance for phytoextraction feasibility. However, high translocation factors were achieved only for some metals after the application of EDTA and high doses of EDDS. Interestingly, in experiments with multielement polluted soil, transfer factors for all metals in *Brassica* species were significantly lower than 1, indicating that the metals were predominantly accumulated in the roots [90]. Based on the obtained results, the authors concluded that phytoextractions of multielement contaminated soils with *Brassica* species is highly limited by concentrations of metals in the soil.

## 6. Enhancing Phytoextraction Using Soil Amendments and Different Planting Strategies

The capacity of *Brassica* species for application in the phytoextraction process can be significantly enhanced by various agricultural techniques, such as the addition of fertilizers, organic manures, or biochar. Considering that phytoextraction is usually associated with heavily polluted and nutrient-poor soils, the addition of fertilizers can significantly improve soil quality, plant growth, and microbial communities. Moreover, fertilizers reduce pH values, thus increasing metal availability, while rapid biomass production can allow for successful extraction of the target element from the soil. The Cd phytoextraction potential of *B. napus* under the influence of eight different types of N fertilizers was investigated by [119,120,121]. The results indicated that the physicochemical acidic N fertilizers increased metal extraction to a greater extent (due to their higher bioavailability at lower pH), even on the poorly contaminated soils, while the physicochemical alkaline N fertilizers proved to be much less effective, with the most significant impact of Ca(NO_3_)_2_ on the extracted Cd concentration. The use of phosphate amendments in soils, particularly in metal-polluted soils, proved to be quite effective in the phytoremediation process [80]. Although they predominantly decrease metal bioavailability [122], positive effects of these amendments on the uptake of As were also observed [123]. A pot experiment with *B. juncea* and *B. napus* under As stress revealed that at a concentration of 100 mg/kg, phosphate significantly improved phytoextraction of As, particularly in *B. napus*. Moreover, this treatment enhanced plant growth, chlorophyll content, and gas exchange parameters, with As uptake and dry biomass of *B. napus* doubled compared to *B. juncea* [123].

The use of organic manure and biochar has also been shown to be advantageous for metal extraction. Mishra et al. [114] have shown that green-manure coapplied with metal-solubilizing bacteria favors the uptake of Cd, Zn, and Pb by *B. juncea*. This addition makes not only for a cheaper method of metal extraction compared to EDTA, but also does not cause biomass reduction (which is one of the major problems with EDTA application) and improves soil conditions. Cárdenas-Aguiar et al. [124] investigated the effect of the addition of manure waste and corresponding biochar prepared under different conditions on Zn uptake by *B. napus*. These amendments improved the soil biochemical characteristics but without influencing plant biomass production. At one site, increased Zn accumulation in roots was observed under the influence of manure biochar prepared at 600 °C and hydrochars prepared at 240 °C. Moreover, according to their results, Zn uptake in *B. napus* plant tissues is soil-related, as opposite accumulation patterns were observed in two mining sites. However, positive effects of manure and biochar application on the uptake of As were detected in both sites. The beneficial effects on *Brassica napus* biomass production were observed when rabbit manure was added to the multicontaminated soil from the Riotinto mining area in Spain, suggesting the feasibility of biochar application in soil loaded with high amounts of different metals [125]. Manure biochar resulted in an increase in heavy metal concentrations in most plant samples, both in roots and shoots, with the most pronounced improvements in the extraction of As and Zn in roots and of As and Se in shoots. Positive effects were also observed in the metal extraction of three *Brassica* species (*B. alba*, *B. carinata*, and *B. nigra*) growing on multicontaminated soil in Italy under the influence of compost and *Bacillus licheniformis* BLMB1, both alone and in combination [126]. In *B. alba* treated with 10% compost and 10% *Bacillus licheniformis* BLMB1 separately, chromium concentrations exceeded the nominal hyperaccumulation threshold (1000 mg/kg) [127]. However, due to the low values of the bioconcentration factors (i.e., lower than 1), these three *Brassica* species can only be successfully implemented in the phytoextraction of soils contaminated with small amounts of metals.

Alteration in cropping regimes may also be beneficial in addressing soil pollution problems. Particularly positive effects on heavy metal extraction have been associated with intercropping, which is considered one of the key factors in metal uptake by plants, along with HM availability [128]. Using this method, non-accumulator species can also be successfully applied in the process of phytoextraction. There are several ways to achieve this outcome, such as influencing the rhizosphere microbiota (by increasing biomass and activity) and using species with different rooting characteristics or different shade tolerances, or species that make more efficient use of water and nutrients [128,129,130]. In addition to the increased uptake of HMs from the soil and faster transport to the aboveground organs, several other positive side-effects, such as a biomass enlargement and more rapid growth, have also been observed [128].

The association of two *Brassica* species (*B. oleracea* var. *acephala sebellica* and *B*. *oleracea* var. *capitata*) with the tree species *Populus alba* (poplar) has shown satisfactory results in removing Cd, Zn, and caffeine from water. After 15 days of exposure, the efficiency of the analyzed group of plants in the removal of Cd, Zn, and CFN was 79–99%, indicating the importance of using plants with significant differences in root characteristics in the elimination of persistent pollutants from water. Moreover, a positive effect of the analyzed heavy metals was observed on the growth of root length of *P*. *alba*, measured by the ratio of root mass and dry weight, while this effect was absent in the *Brassica* cultivars. However, in contrast to poplar, an increase in the ratio of leaf mass was observed in the studied species of the genus *Brassica* [131]. The removal of Cu, Mn, Pb, and Zn from contaminated soils by *B. carinata* was tested under different planting patterns (monoculture, co-planted, or in succession with *Hordeum vulgare*) and showed that an alteration in planting strategy can influence the content of metal in *B. carinata* [132]. Intercropping resulted in a 1.5-fold higher metal accumulation in *B. carinata* compared to monoculture and a reduction in aboveground biomass also, pointing to the presence of competition with *Hordeum vulgare*. Although the observed differences in metals taken up by *B. carinata* in the planting patterns applied in this study were not conspicuous, this species showed that it is capable of growing on multi-metal-contaminated sites and can be used in the naturally assisted remediation of contaminated sites. Cao et al. [130] investigated the effects of intercropping on Cd accumulation in *B. juncea* and *B. napus* grown experimentally with *Sedum alfredii*—a Zn and Cd hyperaccumulator. The results of this study showed that the accumulation of Cd in the shoots of *B. napus* increased by 370% compared to the monoculture when intercropping was applied, while this improvement was much less conspicuous in *B. juncea* (only 27.8%). This effect is achieved through changing the structure of the bacterial communities in the rhizosphere of *B. napus*, which affects metal availability and thus Cd adoption. However, intercropping with *Brassica* species may lead to opposite effects, i.e., reduction in the uptake of the target element. The study conducted by Martínez-Alcalá et al. [133] showed that Zn uptake in *B. juncea* and *Noccaea caerulescens* was significantly higher in monoculture compared to intercropping. The reason for this result is the competition between these two species in Zn uptake as well as their incompatibility in pH values optimal for growth, so crop rotation instead of intercropping could be beneficial for Zn extraction. On the other hand, co-cultivation of *N. caerulescens* with *Lupinus albus* as a metal excluder has been shown to increase Zn accumulation in *Noccaea* and thus has some potential for application in the phytoextraction process.

## 7. The Fate of Harvested Biomass

After the phytoremediation process, produced and harvested biomass has to be disposed of and/or processed. If the biomass generated by phytoremediation can be adequately valorized, the economic viability of the whole process could be enhanced and certain shortcomings that prevent wider use of phytoremediation technologies, such as the long period required for phytoremediation or disposal problems, could be overcome [134]. When the concentration of pollutants in biomass exceeds certain threshold values, it is considered potentially hazardous waste. Therefore, harvested biomass from remediation projects must be adequately managed in order to prevent potential secondary pollution [135]. Research concerning the treatment of harvested biomass is ongoing and aims to find appropriate ways to treat and store this waste with minimal or no hazard to the environment. Several disposal and treatment practices are currently investigated, such as composting, compaction, pyrolysis, leaching, combustion, and gasification [136].

Composting treatment is currently accepted mainly as a volume reducing method in the case of post-remediation contaminated biomass and should be considered only as a pre-treatment. Moreover, leaching tests performed on the composting material showed that dissolved organic matter may increase the mobilization of heavy metals [137]. Research by Krueger et al. [138] on the disposal of biomass after chelate-assisted phytoremediation of Pb with *B. rapa* showed that although a significant reduction of biomass waste material was noticed (within one month of composting a 90% reduction in volume and 50% reduction in mass was achieved) and the composted biomass decreased the amount of water-extractable Pb in the form of Pb–EDTA from 92 to 79%, further treatment of the biomass to lower environmental risk was necessary. The compaction of the harvested biomass after the phytoremediation process results in a product made by pressure, in which process leaching also occurs. However, the results of such investigations and the validations of compacted biomass are still very scarce, and the final product may still be a hazardous waste [139].

Biomass thermal conversion (including pyrolysis, combustion, gasification, and liquefaction) represents one of the most investigated approaches concerning contaminated biomass disposal so far. Generating energy or by-products that could be further used during the conversion of biomass represents a potential advance in this set of methods which could make the phytoremediation biomass management process sustainable and economically viable.

Pyrolysis presents thermal conversion of material in an inert atmosphere in the temperature range of 200–800 °C, resulting in the generation of liquid, gaseous, and solid phases. By optimizing different parameters (pyrolysis device, temperature and heating rate, addition of catalyzers, etc.) it is possible to influence the amount of generated gaseous, liquid, and solid products, as well as the metal concentrations in them [138,140]. Similarly, the content of cellulose, hemicellulose, and lignin, as well as the moisture content of the biomass feedstock, influence the quality and quantity of the final products [141]. Research has mainly tended to increase the concentration of contaminants in the solid phase (biochar) which could afterwards be potentially utilized as a soil conditioner or safely deposited as non-hazardous waste. Recovery of metals concentrated in the biochar by chemical or metallurgical processes would additionally contribute to the economic feasibility of the phytoremediation process [142]. Investigations of biochar from plants used in the phytoremediation process showed that in certain cases of increased metal concentration it could pose a potential environmental risk. Biochar produced from *B. juncea* biomass contaminated with Cd, Pb, and Zn showed that an increased pyrolysis temperature promoted the stabilization of metal fractions in the biochar by decreasing the soluble fraction and increasing the oxidizing and residual fractions [143]. Moreover, further evaluation of biochar ecotoxicity was conducted with *B. rapa* seeds and confirmed decreased ecotoxicity with the elevation of pyrolysis temperature above 550 °C. Similarly, when *B. napus* biomass from soil artificially contaminated with Cd, b, and Zn with and without the addition of EDTA was pyrolyzed at 500 °C most of the metal content remained in the biochar [144]. The stabilization of metals was assessed using toxicity levels characteristic of the leaching procedures associated with municipal solid waste treatment, and revealed values lower than a quantified limit, therefore characterizing this biochar as safe for disposal. Phytoremediation experiments on mixed Cd, Pb, and Zn contaminated sludge and soil with the biomass of *B. rapa* in the presence of an EDTA chelating agent showed a similar trend [145]. After pyrolysis, most of the metal content was concentrated in the solid product: 98.8% for Pb, 97.9% for Cd, and 97.5% for Zn. The results of the leaching tests according to the national legislation showed values lower than the limits, suggesting that the solid product can be safely landfilled. Environmental risk of Cd-contaminated residues (biochar) of *B. napus* at a pyrolysis temperature of 400–700 °C was assessed by Zhang et al. [146]. As the pyrolysis temperature increased, the enrichment factor of Cd in *B. napus* biochar decreased from 2.21 to 1.79. With increasing temperature, the percentage of Cd remaining in the solid phases decreased from 60.15 to 33.53% and migrated to the oxide and residual phases. The results of the leaching toxicity tests showed that a temperature above 600 °C was optimal for deriving biochar with low and acceptable values of Cd according to US EPA limit values and the environmental risk assessment indicators of heavy metals (geological accumulation index, risk assessment index, and potential ecological risk index). Although some research showed promising results, there is a need for further research into stabilizing metals in the solid fraction and filtering and purifying gaseous and liquid fractions derived from the pyrolysis of contaminated biomass. Biomass combustion represents a widely accepted technology for energy production, using high temperature reactions in the presence of oxidizing agents. Heavy metals present in biomass can be retained in bottom ash (solid phase), or in fly ash and flue gasses (gaseous fractions). Certain metals are more volatile (e.g., Cd, Pb, and Zn) and tend to vaporize or re-condense on fly ash particles, while other metals remain in bottom ash [147]. The combustion of *B. juncea* shoots from the field experiments performed at heavy metal and uranium contaminated sites concerned with thermal utilization of phytoremediation biomass showed that the metals and radionuclides were mainly concentrated in the digested sludge from the biogas process, and that 17,000 kJ/kg of bioenergy could be generated during the thermal conversion process [148]. For the successful combustion of biomass, it is preferable to use biomass with a low moisture content, obtain adequate mixing with an oxidizing agent, and to optimize time for the oxidation process [134]. Classic firing systems used for biomass combustion are not suited to burn contaminated biomass, and the use of certain air clearing systems, or adjusted combustion systems, is therefore required [92]. Co-combustion of biomass with coal or sewage sludge is recognized as an additional option, as it could potentially reduce the environmental impacts associated with fossil fuels in terms of reducing greenhouse gas emissions and enhancing the role of bioenergy in total energy production [149,150]. However, the fate of contaminants in combustion products and the degree of their dissemination into the environment requires further study, and the development of technological solutions for overcoming such problems is also needed [151].

Plant biomass can also be converted into liquid phases named biofuels, such as bioethanol and biodiesel, which are potential substitutes for conventional fuels. The production includes (i) pretreatment consisting of hemicellulose removal, (ii) hydrolysis of the cellulose to produce sugar and (iii) the conversion of the sugars to ethanol. Dhiman et al. [152] evaluated the potential of *B. napus* biomass, used for phytoremediation of artificially contaminated soils containing Zn, As, Pb, and Ni at concentrations ranging from 100 to 2000 mg/kg of soil. To test the suitability of contaminated *B. napus* biomass for bioethanol production, a lignocellulosic cocktail from a fungal consortium was tested for enhanced hydrolysis of the biomass. Its resistance to the presence of metals was additionally tested, showing inhibitory effects in the case of Pb, As, and Cd. Higher resistance to the presence of Cu and Ni was also registered, while saccharification yields of 74.4% and 71.8% were obtained with Cu- and Ni-contaminated biomass, respectively. To assess the viability of *B. napus* clear hydrolysate after saccharification, the hydrolysate from the stem (showing the highest saccharification yield and the lowest residual lignin in comparison to other plant parts) was tested for bioethanol production. The results showed that 68.9% of sugar conversion was obtained at an ethanol concentration of 7.6 g/L, which was very similar to the value obtained from uncontaminated biomass (7.7 g/L), pointing to the high efficiency of *B. napus* biomass from phytoremediation processes for bioethanol production.

Biodiesel is produced from oils extracted from plant seeds that have undergone several modification processes, among which the most widespread is known as transesterification and is based on the use of catalysts [153]. After such a process, biodiesel can be used in standard diesel engines. Recently, the non-catalyzed supercritical methanol process has been recognized for its higher production rate and for its reduction of used energy, with less waste generated in the process [154]. The main problem to be addressed in the use of biomass from phytoremediation sites for biodiesel production is the transfer of contaminants from the biomass to the oil. Investigations by Angelova et al. [155] found that the contents of Pb, Cu, and Cd in *B. napus* oil originated from biomass grown on a contaminated industrial site were six to nine times the concentration permitted under national legislation. Contrary to that, Park et al. [34] investigated the feasibility of oil extraction from *B. napus* grown on Cu, Zn, Cd, Pb, and As contaminated areas in the vicinity of a copper smelter site. Although *B. napus* showed an increased rate of metal accumulation from the contaminated site, it was found that more than 50% of the metal content remained in the residues of seeds after the extraction of oil, making biodiesel production from such biomass an environmentally acceptable option. Saka and Kuzdiana [156] reported considerably shorter reaction times and higher yields of biodiesel from *B. napus*, along with simplified purification of the product by using a supercritical methanol process. However, the issue of metal transfer from the plant to the final product requires further and more detailed research to assess the issue of metal content in generated biodiesel and in the exhaust fumes.

Biogas is produced by the anaerobic digestion of biomass by microorganisms which break down organic matter and produce carbon dioxide and methane as by-products. Biogas is considered an environmentally friendly product that can be used for heating and electricity production. An assessment of biogas production from biomass through phytoremediation processes involving metal-accumulating and -hyperaccumulating plants revealed that microorganisms included in anaerobic digestion had different responses to metal concentration in biomass [157]. An increased content of Cu (up to 1000 mg/kg) and Mn (up to 10,000 mg/kg) promoted anaerobic digestion, while larger concentrations of Mn (up to 20,000 mg/kg) and Zn (above 500 mg/kg) inhibited the anaerobic digestion process. Additionally, the presence of Cd (up to 200 mg/kg), Pb (up to 2000 mg/kg), and As (up to 10,000 mg/kg) showed no change in cumulative biogas production. This research showed that metal-accumulating plants from phytoextraction processes may be suitable for biogas production, particularly in the case of soils contaminated with Cu, Pb, Cd, and As. Similarly, shoots of *B. napus* contaminated with Cu were tested for biogas production and the results showed that the cumulative methane production of biomass with 100, 500, 1000, and 5000 mg/kg Cu was 8%, 12.3%, 14.6%, and 41.2% lower compared to the control group with low Cu content, respectively [158]. It has been revealed that biomass containing 100 mg/kg Cu actually shortened digestion time and promoted anaerobic digestion, while the concentration of 500–5000 mg/kg Cu treatments reduced the cumulative biogas production from 8630 to 5783 mL, showing that higher concentrations of Cu in biomass (>500 mg/kg) hinder anaerobic digestion of *B. napus*. Biogas production from shoots of *B. juncea* grown at a site contaminated with Al, Co, Zn, Ni, and U was successful, and no retardation of biogas formation was noticed in comparison to *B. juncea* biomass from an uncontaminated site [148]. However, to date, experimental data on anaerobic digestion of heavy-metal-contaminated plants from the Brassicaceae family remain scarce such that further investigations are needed.

There is still no established method for dealing with biomass residues from the phytoremediation process. An approach that integrated phytoremediation with the utilization of harvested biomass for energy or metal production in a sustainable manner would provide a way towards more environmentally and economically viable solutions, which would additionally enhance the general acceptance of the phytoremediation method. The mechanisms to avoid the release of toxic compounds into the environment are yet to be determined, and techniques for their reuse need to be established. Concentrations of metals in fewer fractions, followed by the optimization of processing parameters, should be coupled with the utilization of these fractions as a source of reusable materials [154]. Post-remediation management of biomass should also be appropriately regulated by separate or existing laws to address the safe and efficient use and deposition of harvested biomass [135]. The Life Cycle Assessment process can help identify environmental aspects and bottlenecks of the phytoremediation process and assess its products throughout the life cycle. If the optimization of performances is reached and safe by-products are generated, such an approach could deliver more environmental and economic benefits than the accepted energy-oriented practice [159]. The energy efficiency of the phytoremediation process should also be determined, taking into account total energy inputs and outputs in field studies over several vegetation seasons in order to enhance the sustainability of the process [160].

## 8. The Challenges of Moving from Pot to Field Experiments

Based on the results of many studies and experiments conducted in the last decade, it can be concluded that *Brassica* species have significant potential to be used in phytoextractions. However, the estimation of their potential has primarily been made based on the results of pot experiments such that their real field potential and the feasibility of the whole process of phytoextraction under authentic natural conditions have not yet been assessed.

To date, there are only a limited number of studies in which the results of field trials with *Brassica* species are reported [14,76,161]. Only one experiment, conducted by Bruneti et al. [78], aimed to assess the difference between metal uptake of *B. napus* grown on polluted soil in greenhouse conditions and in field conditions. The authors concluded that while *B. napus* accumulated relatively high amounts of metals in greenhouse conditions, it failed to take up the same amounts of metals when grown on field, probably due to weather and other site-specific conditions. Other field studies focused primarily on assessing the suitability of different *B. napus* and *B. juncea* genotypes for phytoextractions of soils polluted with Cd and Pb [14,161]. The authors grew 80 different *B. juncea* and 28 *B. napus* genotypes to full maturity. They observed significant differences between Cd and Pb uptake and in translocation in aboveground parts between different genotypes, indicating that there are preferred genotypes suitable for Cd and Pb phytoextraction in low to moderately contaminated soils.

**Figure 1:**
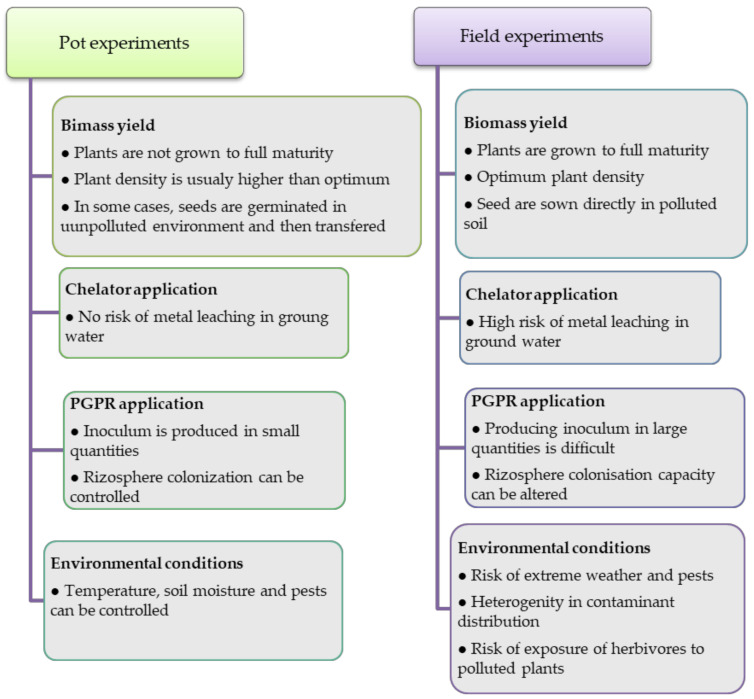
Main differences in growing conditions in pot and field experiments.

Compared to pot tests, field experiments are performed under different conditions which might affect not only plant performance and the efficiency of phytoextraction but may also have an impact on the surrounding environment. Figure 1 summarizes the main differences in growing conditions in pot and field experiments.

Biomass yield plays an important role in phytoextraction since, beside metal concentration in plants, it is one of the key parameters in efficient metal extraction from soil. In order to assess the real potential of plants grown in polluted soils to produce sufficient biomass, they should be grown to full maturity. This is especially important when oil crops are grown, as the oil produced from the seeds can be used for biofuel production [152,156,160]. However, in most of the pot experiments the growth period of *Brassica* plants was 60 days or shorter, which is not enough for plants to reach maturity [30,76,97,98,99,101,103,104,112,113,115,120,123,124,125]. Another factor influencing biomass yield is plant density. In the field, plant density is optimized for a maximum yield of grown plant species. However, in pot experiments, the number of plants is often higher than is optimal, which affects plant growth and biomass yields. In several studies, a small amount of soil, equal or less than 0.5 kg, was used [103,113,115,124,125,133] for growing three [103] or ten plants [124]. Other studies reported higher quantities of polluted soil used for pot experiments. Most frequently, 2–5 kg of soil per pot was used in experiments [76,98,104,106,112,116], but the number of plants grown is usually three or five, which is still higher than optimal. In several experiments, seeds of *Brassica* species were not sown directly in the contaminated soil. Plants were first germinated for a week or two in sterilized and humidified vermiculite [115], sand [133], or unpolluted substrate [116,162] and then transferred to pots with contaminated soil. Germination of the plants in ab unpolluted environment helps their development in the early, most sensitive phase of growth and ensures that strong and healthy plants are used for the experiments. However, in real field conditions, seeds are sown in polluted soils and heavy metals can cause stress which affects seed germination, seedling growth, and overall biomass production. Thus, the results of experiments with pre-germinated plants must be analyzed with caution.

Chemically or biologically enhancing the uptake of heavy metals by plants is a promising strategy for increasing phytoextraction efficiency, but the use of such modifications also has certain disadvantages. The application of chelating agents in polluted soil can increase the risk of heavy metal leaching and spread the contaminants. The chelate concentrations required to induce significant metal uptake by shoots are usually high (4–8 mmol/kg). However, only a small amount of the mobilized metals can be absorbed by the plants. The rest of the mobilized metals, together with the chelator, stay in the soil and can contaminate ground water by leaching. EDTA is one of the most effective chelators, but EDTA–metal complexes have very low biodegradability, and the risk of leaching is extremely high. Besides that, as EDTA is one of the most common surface water contaminants, its application to soil is restricted in some countries, especially in Europe [163]. Due to the high risk of metal leaching, chelant-assisted phytoextraction should only be performed when there is no connection to the groundwater (e.g., ex situ).

PGPR suitable for enhancing phytoextraction have to be tolerant to high metal concentrations, are usually very specific, and often must be isolated from the rizosphere of plants already growing in a polluted site [97,98,101,162]. Producing higher quantities of inocula suitable for field application is a challenge. PGPR inocula must have a long shelf-life and a high rizosphere colonization capacity under field conditions. The colonization capacity may decrease due to competition with resident soil microorganisms or due to the application of fumigants which alter the microbial structure of the soil. All these factors can influence the feasibility of the PGPR-enhanced phytoextraction process.

Based on the studies published on the suitability of *Brassica* species for phytoextraction, it can be concluded that these species have significant potential for use in soil remediation. However, future experiments must be scaled up to the field, with special attention paid to all site-specific factors that may affect the feasibility of the process with respect to environmental concerns.

So far, the most promising results have been obtained with PGPR application, which increased metals uptake by up to 60% with no reduction of biomass [97,98,99,102,103,104,105]. Further research should therefore be directed towards conducting field tests that can provide valuable information about complex plant–microbe–metal interactions under natural conditions, which presents the next step towards the practical application and commercialization of PGPR products. The addition of organic amendments, such as biochar and manure, has been shown to have a positive effect on heavy metal uptake by *Brassica* species (uptake by shoots was increased by up to 30% [114,124,125,126] without influencing biomass production). Field experiments are needed to further investigate the long-term effectiveness of organic amendments under natural conditions which involve temperature variations, heterogeneity in soil properties, and different cropping strategies. Moreover, research combining biologically or chemically assisted phytoremediation with the addition of organic amendments could contribute significantly to the development of successful remediation practices. Finally, harvested biomass containing pollutants may pose a considerable risk to the environment if not properly handled. Research concerning the treatment of harvested biomass should progress simultaneously with phytoextraction experiments in order to develop realistic solutions for the practical application of phytoremediation technologies using *Brassica* species.

## Figures and Tables

**Table 1:** Examples of PGPR/PGPE application in phytoremediaton with *Brassica* species.

PGPR/PGPE	Plant	Metal	Conditions	PGPR/PGPE Effect	Reference
*Bacillus sp*. PZ-1	*Brassica juncea*	Pb	Pot experiment/ spiked soil	Increased biomass (up to 35%) Increased Pb uptake by shoots (52–106%) and roots (28.3–83.6%) Increased TFroot-shoot (12–55%)	[99]
*Bacillus toyonensis* (MG430287) *Rhodococcus hoagii* (MG432495) *Lysinibacillus mangiferihumi* (MG432492) *Lysinibacillus fusiformis* (MG430290)	*Brassica juncea*	Fe	Pot experiment/ contaminated soil	Increased rot length (47–106%) Increased shoot length (49–71%) Increased Fe uptake (57.91–128%) Increased biosynthesis of antioxidant molecules	[101]
*Bacteroidetes bacterium*, *Pseudomonas fluorescens Variovorax* sp.	*Brassica napus*	Cd, Cu, Pb, Zn	Pot experiment/ contaminated soil	No increase in biomass Increased Cd uptake by roots (up to 12%) and shoots (up to 10%) Increased Zn uptake (18% in shoots, 8% in roots)	[102]
Isolates SMV242, SMV244, SMV248, SMV250, and SMV251 belonging to the following three phyla: Actinobacteria, Proteobacteria, and Firmicutes	*Brassica juncea*	As	Pot experiment/ contaminated soil	No increase in biomass Increased As uptake only in roots (55%) Increased As uptake by shoots only in the presence of mobilizing agent K_2_HPO_4_ (150%)	[98]
*Burkholderia phytofirmans* PsJN^T^	*Brassica juncea*	Zn, Pb, Cd, Cu	Pot experiment/ contaminated soil	No increase in biomass Increased shoot uptake of Cd (22%) and Zn (38%)	[104]
*Variovorax. paradoxus* strain 5C-2, *Rhizobium leguminosarum* bv. i strain RCAM1066, AMF strain *Glomus* sp. 1Fo	*Brassica juncea*	Cd	Pot experiment/ spiked soil	No increase in biomass Increased Cd uptake (up to 10%)	[105]
*Serratia* K120, *Enterobacter* K125, *Serratia* MC107, *Serratia* MC119 and *Enterobacter* MC156	*Brassica juncea*	As, Cd, Cu, Cr, Pb	Pot experiment/ contaminated soil	Increased stem height (up to two times) Increased root length (up to five times) Increased metal uptake by shoots and roots (up to 1072 mg/kg of Pb in shoots and 1815 mg/kg Pb in roots)	[97]
*Burkholderia* sp. SaMR10 *Burkholderia* sp. SaZR4 *Sphingomonas* sp. SaMR12 *Variovorax* sp. SaNR1	*Brassica napus*	Cd	Field experiment/ contaminated soil	Increased yield and biomass (6–7.8%) Increased Cd uptake by shoots (47%) and roots (57%) Increased TF root-shoot (59%) and TF shoot-pod (10%)	[102]
*Fusarium* sp. CBRF44, *Penicillium* sp. CBRF65	*Brassica napus*	Cd, Pb	Pot experiment/ spiked soil	Increased biomass (32–47%) Increased Pb mobility in soil (up to 83%) Increased Cd (20–60 %) and Pb (15–46%) uptake	[103]
*Alternaria* sp. CBSF68	*Brassica napus*	Cd, Pb	Pot experiment/ spiked soil	No increase in metal uptake	[103]

**Table 2:** The influence of chelator application on biomass reduction in *Brassica* plants.

Plant	Metal	Time of Application	Chelator	Biomass Reduction	Reference
*Brassica juncea*	Cd, Cr, Cu, Ni, Pb, Zn (spiked soil)	1 week before harvesting	EDDS (5 mmol/kg)	40%	
			CA (10 mmol/kg)	27%	[89]
			GLDA (3 mmol/kg)	No reduction	
*Brassica rapa*	Cd, Cr, Cu, Ni, Pb, Zn (spiked soil)	1 week before harvesting	EDDS (5 mmol/kg)	13%	
			CA (10 mmol/kg)	18%	[89]
			GLDA (3 mmol/kg)	No reduction	
*Brassica napus*	Cu	4 weeks before harvesting	EDDS (2 mmol/kg)	No reduction	[111]
			EDDS (4 mmol/kg)	27%	
			EDDS (8 mmol/kg)	65%	
			EDTA (2 mmol/kg)	No reduction	
			EDTA (4 mmol/kg)	22%	
			EDTA (8 mmol/kg)	21%	
*Brassica juncea*	Cd, Zn	30 days before harvesting	EDTA (5 mmol/kg)	58.4%	[112]
			EDTA (10 mmol/kg)	72.6%	
*Brassica juncea*	Pb	1 week before harvesting	EDTA (2.5 mmol/kg)	up to 37.5%	[115]
			CA (25 mmol/kg)	up to 20.2%	
*Brassica oleracea*	Pb (spiked soil)	No data	EDTA (5 mmol/kg)	Up to 20.22%	[116]
	Zn (spiked soil)		EDTA (5 mmol/kg)	Up to 16.21%	

**Table 3:** Metal concentration in shoots, bioconcentration and translocation factors for *Brassica* plants after chelator application.

Plant	Metal	Chelator	Metal Concentration in Shoots (mg/kg)	BCF	TF	Reference
*Brassica juncea*	Cd, Cr, Cu, Ni, Pb, Zn (spiked, multielement)	No chelator	Cr: 20; Zn: 60; Cd: 20; Pb: 200; Ni: 100; Cu: 20	Cr: 0.64; Zn: 1.57; Cd: 1.93; Pb: 1.57; Ni: 2.17; Cu: 0.53	<1 for all metals	[89]
		EDDS (5 mmol/kg)	Cr: 70; Zn: 120; Cd: 60; Pb: 300; Ni: 170; Cu: 40	Cr: 1.44; Zn: 2.68; Cd: 3.36; Pb: 2.45; Ni: 3.69; Cu: 0.87	<1 for all metals	
		CA (10 mmol/kg)	Cr: 57; Zn: 90; Cd: 40; Pb: 300; Ni: 100; Cu: 35	Cr: 1.45; Zn: 2.59; Cd: 2.78; Pb: 2.25; Ni: 4.01; Cu: 0.77	<1 for all metals	
		GLDA (3 mmol/kg)	Cr: 60; Zn: 100; Cd: 40; Pb: 300; Ni: 170; Cu: 40	Cr: 1.25; Zn: 2.53; Cd: 2.45; Pb: 2.03; Ni: 3.24; Cu: 0.77	<1 for all metals	
*Brassica rapa*	Cd, Cr, Cu, Ni, Pb, Zn (spiked, multielement)	No chelator	Cr: 20; Zn: 80; Cd: 20; Pb: 120; Ni: 80; Cu: 20	Cr: 0.68; Zn: 1.83; Cd: 2.17; Pb: 1.28; Ni: 2.29; Cu: 0.59	<1 for all metals	[89]
		EDDS (5 mmol/kg)	Cr: 40; Zn: 110; Cd: 35; Pb: 180; Ni: 80; Cu: 20	Cr: 1.06; Zn: 2.62; Cd: 3.65; Pb: 1.74; Ni: 2.90; Cu: 0.62	<1 for all metals	
		CA (10 mmol/kg)	Cr: 60; Zn: 80; Cd: 33; Pb: 170; Ni: 75; Cu: 15	Cr: 1.24; Zn: 2.38; Cd: 3.01; Pb: 1.52; Ni: 2.94; Cu: 0.47	<1 for all metals	
		GLDA (3 mmol/kg)	Cr: 57; Zn: 90; Cd: 40; Pb: 300; Ni: 100; Cu: 35	Cr: 1.26; Zn: 2.62; Cd: 3.43; Pb: 1.58; Ni: 2.04; Cu: 0.44	<1 for all metals	
*Brassica napus*	Cu	No chelator	16.6	No data	<1	[111]
		EDDS (2 mmol/kg)	38.6	No data	<1	
		EDDS (4 mmol/kg)	131.5	No data	>1	
		EDDS (8 mmol/kg)	316.4	No data	>1	
		EDTA (2 mmol/kg)	34.2	No data	<1	
		EDTA (4 mmol/kg)	51.5	No data	<1	
		EDTA (8 mmol/kg)	52.0	No data	<1	
*Brassica juncea*	Cd, Zn	No chelator	Cd: 11 Zn: 160	Cd: 0.78 Zn: 0.28	<1 for Cd and Zn	[112]
		EDTA (5 mmol/kg)	Cd: 16 Zn: 410	Cd: 1.41 Zn: 0.65	>1 for Cd <1 for Zn	
		EDTA (10 mmol/kg)	Cd: 18 Zn: 420	Cd: 1.70 Zn: 0.72	>1 for Cd <1 for Zn	
		EDTA (5 mmol/kg) + CA (5 mmol/kg)	Cd: 12 Zn: 380	Cd: 1.08 Zn: 0.52	>1 for Cd <1 for Zn	
		EDTA (5 mmol/kg) + CA (10 mmol/kg)	Cd: 11 Zn: 400	Cd: 1.03 Zn: 0.48	<1 for Cd and Zn	
		EDTA (5 mmol/kg) + OA (5 mmol/kg)	Cd: 16 Zn: 400	Cd: 1.31 Zn: 0.58	<1 for Cd and Zn	
		EDTA (5 mmol/kg) + OA (10 mmol/kg)	Cd: 12 Zn: 300	Cd: 0.94 Zn: 0.48	<1 for Cd and Zn	
*Brassica juncea*	Pb	No chelator	5	0.018	<1	[115]
		EDTA (2.5 mmol/kg)	45	0.134	>1	
*Brassica oleracea*	Pb, Zn	No chelator	Pb: nd Zn: nd	No data	No data	[117]
		EDTA (5 mmol/kg)	Pb: 20.62 Zn: 42.58	No data	No data	

## Data Availability

The data presented in this study are available in article.

## References

[1] Suresh B., Ravishankar G.A. (2004). Phytoremediation—A Novel and Promising Approach for Environmental Clean-up. Crit. Rev. Biotechnol..

[2] Susarla S., Medina V.F., McCutcheon S.C. (2002). Phytoremediation: An ecological solution to organic chemical contamination. Ecol. Eng..

[3] Wang L., Ji B., Hu Y., Liu R., Sun W. (2017). A review on in situ phytoremediation of mine tailings. Chemosphere.

[4] Rascio N., Navari-Izzo F. (2011). Heavy metal hyperaccumulating plants: How and why do they do it? And what makes them so interesting?. Plant. Sci..

[5] Pandey V.C., Rai A., Korstad J., Pandey V.C., Bauddh K. (2019). Aromatic crops in phytoremediation: From contaminated to waste dumpsites. Phytomanagement of Polluted Sites.

[6] Zhang Y., Li C., Ji X., Yun C., Wang M., Luo X. (2020). The knowledge domain and emerging trends in phytoremediation: A scientometric analysis with CiteSpace. Environ. Sci. Pollut. Res..

[7] Khan I., Iqbal M., Shafiq F. (2019). Phytomanagement of lead-contaminated soils: Critical review of new trends and future prospects. Int. J. Environ. Sci. Technol..

[8] Yan A., Wang Y., Tan S.N., Yusof M.L.M., Ghosh S., Chen Z. (2020). Phytoremediation: A Promising Approach for Revegetation of Heavy Metal-Polluted Land. Front. Plant Sci..

[9] Ghori Z., Iftikhar H., Bhatti M.F., Sharma I., Kazi A.G., Ahmad P., Ahmad P. (2015). Phytoextraction: The use of plants to remove heavy metals from soil. Plant Metal Interaction: Emerging Remediation Techniques.

[10] Favas P.J., Pratas J., Varun M., D’Souza R., Paul M.S., Hernandez-Soriano M.C. (2014). Phytoremediation of soils contaminated with metals and metalloids at mining areas: Potential of native flora. Environmental Risk Assessment of Soil Contamination.

[11] Krämer U. (2010). Metal Hyperaccumulation in plants. Annu. Rev. Plant Biol..

[12] Suman J., Uhlik O., Viktorova J., Macek T. (2018). Phytoextraction of heavy metals: A promising tool for clean-up of polluted environment?. Front. Plant Sci..

[13] Ali Z., Waheed H., Gul A., Afzal F., Anwaar K., Imran S., Ahmad P. (2017). Brassicaceae plants: Metal accumulation and their role in phytoremediation. Oilseed Crops: Yield and Adaptations under Environmental Stress.

[14] Gurajala H.K., Cao X., Tang L., Ramesh T.M., Lu M., Yang X. (2019). Comparative assessment of Indian mustard (*Brassica juncea* L.) genotypes for phytoremediation of Cd and Pb contaminated soils. Environ. Pollut..

[15] Drozdova I., Alekseeva-Popova N., Dorofeyev V., Bech J., Belyaeva A., Roca N. (2019). A comparative study of the accumulation of trace elements in Brassicaceae plant species with phytoremediation potential. Appl. Geochem..

[16] Raza A., Hafeez M.B., Zahra N., Shaukat K., Umbreen S., Tabassum J., Charagh S., Khan R.S.A., Hasanuzzaman M., Hasanuzzaman M. (2020). The plant family brassicaceae: Introduction, biology, and importance. The Plant Family Brassicaceae.

[17] Anjum N.A., Gill S.S., Ahmad I., Pacheco M., Duarte A.C., Umar S., Khan N.A., Pereira M.E. (2012). The Plant Family Brassicaceae: An Introduction. The Plant Family Brassicaceae.

[18] Cappa J.J., Yetter C., Fakra S., Cappa P.J., DeTar R., Landes C., Pilon-Smits E.A.H., Simmons M.P. (2014). Evolution of selenium hyperaccumulation in *Stanleya* (Brassicaceae) as inferred from phylogeny, physiology and X-ray microprobe analysis. N. Phytol..

[19] Reeves R.D., Baker A.J.M., Jaffré T., Erskine P.D., Echevarria G., van der Ent A. (2017). A global database for hyperaccumulator plants of metal and metalloid trace elements. N. Phytol..

[20] Dimitrakopoulos P.G., Aloupi M., Tetradis G., Adamidis G.C. (2021). Broomrape Species Parasitizing *Odontarrhena lesbiaca* (Bras-sicaceae) Individuals Act as Nickel Hyperaccumulators. Plants.

[21] Mišljenović T., Jovanović S., Mihailović N., Gajić B., Tomović G., Baker A.J.M., Echevarria G., Jakovljevic K. (2019). Natural variation of nickel, zinc and cadmium (hyper)accumulation in facultative serpentinophytes *Noccaea kovatsii* and *N*. praecox. Plant Soil.

[22] Yang Y., Liang Y., Han X., Chiu T.-Y., Ghosh A., Chen H., Tang M. (2016). The roles of arbuscular mycorrhizal fungi (AMF) in phytoremediation and tree-herb interactions in Pb contaminated soil. Sci. Rep..

[23] Misra S., Misra K.G., Venkatramanan S.S.V., Prasad R. (2019). Phytoremediation: An alternative tool towards clean and green environment. Sustainable Green Technologies for Environmental Management.

[24] (2013). The Plant List. http://www.theplantlist.org.

[25] Nikolić V., Josifović M. (1972). Brassica. Flora Srbije.

[26] Ball P.W., Heywood V.H., Akeroyd J.R., Tutin T.G., Burges N.A., Chater A.O., Edmondson J.R., Heywood V.H., Moore D.M., Valentine D.H., Walters S.M., Webb D.A. (1993). Cruciferae. Flora Europaea.

[27] Murovec J., Guček K., Bohanec B., Avbelj M., Jerala R. (2018). DNA-Free Genome Editing of *Brassica oleracea* and *B. rapa* Protoplasts Using CRISPR-Cas9 Ribonucleoprotein Complexes. Front. Plant Sci..

[28] Mourato M.P., Moreira I.N., Leitão I., Pinto F.R., Sales J.R., Martins L.L. (2015). Effect of Heavy Metals in Plants of the Genus Brassica. Int. J. Mol. Sci..

[29] Farahani M., Naderi R., Mazhari M. (2015). Phytoremediation of Cd contaminated soils by ornamental cabbage (*Brassica oleracea*) species. J. Biodivers. Environ. Sci..

[30] Cojocaru P., Gusiatin Z.M., Cretescu I. (2016). Phytoextraction of Cd and Zn as single or mixed pollutants from soil by rape (*Brassica napus*). Environ. Sci. Pollut. Res..

[31] Napoli M., Cecchi S., Grassi C., Baldi A., Zanchi C.A., Orlandini S. (2018). Phytoextraction of copper from a contaminated soil using arable and vegetable crops. Chemosphere.

[32] Chen L., Long C., Wang D., Yang J. (2019). Phytoremediation of cadmium (Cd) and uranium (U) contaminated soils by *Brassica juncea* L. enhanced with exogenous application of plant growth regulators. Chemosphere.

[33] Anjum N.A., Gill S.S., Ahmad I., Duarte A.C., Umar S., Khan N.A., Pereira M.E., Anjum N.A., Pereira M.E., Ahmad I., Duarte A.C., Umar S., Khan N.A. (2012). Metals and Metalloids Accumulation Variability in *Brassica* Species. Phytotechnologies: Remediation of Environmental Contaminants.

[34] Park J., Kim J.-Y., Kim K.-W. (2012). Phytoremediation of soil contaminated with heavy metals using *Brassica napus*. Geosystem Eng..

[35] Kumar V., Mahajan M., Yadav S.K., Anjum N., Ahmad I., Pereira M., Duarte A., Umar S., Khan N. (2012). Toxic metals accumulation, tolerance and homeostasis in brassicaoilseed species: Overview of physiological, biochemical and molecular mechanisms. The Plant Family Brassicaceae.

[36] Jabeen N., Hasanuzzaman M. (2020). Agricultural, Economic and Societal Importance of Brassicaceae Plants. The Plant Family Brassicaceae.

[37] Bączek-Kwinta R., Antonkiewicz J., Łopata-Stasiak A., Kępka W. (2019). Smoke compounds aggravate stress inflicted on *Brassica* seedlings by unfavourable soil conditions. Photosynthetica.

[38] Haghighi M., Kafi M., Pessarakli M., Sheibanirad A., Sharifinia M.R. (2016). Using kale (*Brassica oleracea* var. *acephala*) as a phytoremediation plant species for lead (Pb) and cadmium (Cd) removal in saline soils. J. Plant Nutr..

[39] Teklehaymanot T., Wang H., Liang J., Wu J., Lin R., Zhou Z., Cai X., Wang X. (2019). Variation in Plant Morphology and Sinigrin Content in Ethiopian Mustard (*Brassica carinata* L.). Hortic. Plant J..

[40] Gatto A.D., Pieri S., Mangoni L., Candilo M.D., Diozzi M., Mastro G.D., Verdini L., Signor M., Barbiani G., Carboni G. (2010). The cultivars of *Brassica carinata* for the next sowing. Inf. Agrar..

[41] Canam T., Li X., Holowachuk J., Yu M., Xia J., Mandal R., Krishnamurthy R., Bouatra S., Sinelnikov I., Yu B. (2013). Differential metabolite profiles and salinity tolerance between two genetically related brown-seeded and yellow-seeded *Brassica carinata* lines. Plant Sci..

[42] Manara A., Fasani E., Furini A., DalCorso G. (2020). Evolution of the metal hyperaccumulation and hypertolerance traits. Plant Cell Environ..

[43] Liu N., Lin Z.-F., Lin G.-Z., Song L.-Y., Chen S.-W., Mo H., Peng C.-L. (2010). Lead and cadmium induced alterations of cellular functions in leaves of *Alocasia macrorrhiza* L. Schott. Ecotoxicol. Environ. Saf..

[44] Nazir M.M., Ulhassan Z., Zeeshan M., Ali S., Gill M.B., Hasanuzzaman M. (2020). Toxic metals/metalloids accumulation, tolerance, and homeostasis in *Brassica* oilseed species. The Plant Family Brassicaceae.

[45] Gill R.A., Zang L., Ali B., Farooq M.A., Cui P., Yang S., Ali S., Zhou W. (2015). Chromium-induced physio-chemical and ultra-structural changes in four cultivars of *Brassica napus* L. Chemosphere.

[46] Ulhassan Z., Ali S., Gill R.A., Mwamba T.M., Abid M., Li L., Zhang N., Zhou W. (2018). Comparative orchestrating response of four oilseed rape (*Brassica napus*) cultivars against the selenium stress as revealed by physio-chemical, ultrastructural and molecular profiling. Ecotoxicol. Environ. Saf..

[47] Chen L., Yang L., Wang Q. (2009). In vivo phytochelatins and Hg–phytochelatin complexes in Hg-stressed *Brassica chinensis* L. Metallomics.

[48] Roychoudhury A., Krishnamoorthi S., Paul R., Wani S., Thakur A., Jeshima Khan Y. (2020). Arsenic toxicity and molecular mechanism of arsenic tolerance in different members of Brassicaceae. Brassica Improvement.

[49] Yadav S. (2010). Heavy metals toxicity in plants: An overview on the role of glutathione and phytochelatins in heavy metal stress tolerance of plants. S. Afr. J. Bot..

[50] Li H., Pu P., Li X., Gong Y., An D., Zhang L., Lv J. (2020). Sulfur application reduces cadmium uptake in edible parts of pakchoi (*Brassica chinensis* L.) by cadmium chelation and vacuolar sequestration. Ecotoxicol. Environ. Saf..

[51] Wu Z., McGrouther K., Chen N., Wu W., Wang H. (2013). Subcellular Distribution of Metals within *Brassica chinensis* L. in Response to Elevated Lead and Chromium Stress. J. Agric. Food Chem..

[52] Ahmad A., Alam M. (2004). Sequestration and remediation of heavy metals by *Brassica* sp. at Hindan River sites. Indian J. Chem. Technol..

[53] Mohamed A., Castagna A., Ranieri A., di Toppi L.S. (2012). Cadmium tolerance in *Brassica juncea* roots and shoots is affected by antioxidant status and phytochelatin biosynthesis. Plant Physiol. Biochem..

[54] Sharma P., Jha A.B., Dubey R.S., Pessarakli M. (2012). Reactive Oxygen Species, Oxidative Damage, and Antioxidative Defense Mechanism in Plants under Stressful Conditions. J. Bot..

[55] Singh S., Sinha S. (2005). Accumulation of metals and its effects in *Brassica juncea* L. Czern (cv. Rohini) grown on various amendments of tannery waste. Ecotoxicol. Environ. Safe..

[56] Ahmad J., Baig M.A., Ali A.A., Al-Huqail A.A., Ibrahim M.M., Qureshi M.I. (2018). Differential antioxidative and biochemical responses to aluminium stress in *Brassica juncea* cultivars. Hortic. Environ. Biotechnol..

[57] Ali M.A., Ashraf M., Athar H.R. (2009). Influence of nickel stress on growth and some important physiological/biochemical attributes in some diverse canola (*Brassica napus* L.) cultivars. J. Hazard. Mater..

[58] Sinha S., Pandey K. (2003). Nickel Induced Toxic Effects and Bioaccumulation in the Submerged Plant, *Hydrilla verticillata* (L.F.) Royle Under Repeated Metal Exposure. Bull. Environ. Contam. Toxicol..

[59] Kraemer U., Cotter-Howells J.D., Charnock J.M., Baker A.J.M., Smith J.A.C. (1996). Free histidine as a metal chelator in plants that accumulate nickel. Nature.

[60] Freeman J., Persans M.W., Nieman K., Albrecht C., Peer W., Pickering I., Salt D.E. (2004). Increased Glutathione Biosynthesis Plays a Role in Nickel Tolerance in *Thlaspi* Nickel Hyperaccumulators. Plant Cell.

[61] Corso M., de la Torre V.S.G. (2020). Biomolecular approaches to understanding metal tolerance and hyperaccumulation in plants. Metallomics.

[62] Hanikenne M., Nouet C. (2011). Metal hyperaccumulation and hypertolerance: A model for plant evolutionary genomics. Curr. Opin. Plant Biol..

[63] Talke I.N., Hanikenne M., Krämer U. (2006). Zinc-Dependent Global Transcriptional Control, Transcriptional Deregulation, and Higher Gene Copy Number for Genes in Metal Homeostasis of the Hyperaccumulator *Arabidopsis halleri*. Plant Physiol..

[64] Van de Mortel J.E., Almar Villanueva L., Schat H., Kwekkeboom J., Coughlan S., Moerland P.D., ver Loren van Themaat E., Koornneef M., Aarts M.G. (2006). Large expression differences in genes for iron and zinc homeostasis, stress response, and lignin biosynthesis distinguish roots of Arabidopsis thaliana and the related metal hyperaccumulator *Thlaspi caerulescens*. Plant Physiol..

[65] Ahmed M., Singh V.K., Upadhyay R.S., Anjum N., Ahmad I., Pereira M., Duarte A., Umar S., Khan N. (2012). Brassica rhizosphere-microbe interactions and their role in phytoremediation. The Plant Family Brassicaceae.

[66] Tamaoki M., Freeman J.L., Pilon-Smits E.A. (2008). Cooperative Ethylene and Jasmonic Acid Signaling Regulates Selenite Resistance in *Arabidopsis*. Plant Physiol..

[67] D’Alessandro A., Taamalli M., Gevi F., Timperio A.M., Zolla L., Ghnaya T. (2013). Cadmium Stress Responses in *Brassica juncea*: Hints from Proteomics and Metabolomics. J. Proteome Res..

[68] Orroño D.I., Lavado R.S. (2009). Distribution of extractable heavy metals in different soil fractions. Chem. Speciat. Bioavailab..

[69] Peng S., Wang P., Peng L., Cheng T., Sun W., Shi Z. (2018). Predicting Heavy Metal Partition Equilibrium in Soils: Roles of Soil Components and Binding Sites. Soil Sci. Soc. Am. J..

[70] Chigbo C., Batty L. (2013). Phytoremediation potential of *Brassica juncea* in Cu-pyrene co-contaminated soil: Comparing freshly spiked soil with aged soil. J. Environ. Manag..

[71] Lock K., Janssen C.R. (2001). Ecotoxicity of Zinc in Spiked Artificial Soils versus Contaminated Field Soils. Environ. Sci. Technol..

[72] Qu C., Chen W., Hu X., Cai P., Chen C., Yu X.Y., Huang Q. (2019). Heavy metal behavior at mineral-organo interfaces: Mechanisms, modelling and influence factors. Environ. Int..

[73] Ma Y., Lombi E., Oliver I.W., Nolan A.L., McLaughlin M.J. (2006). Long-Term Aging of Copper Added to Soils. Environ. Sci. Technol..

[74] Lu A., Zhang S., Qin X., Wu W., Liu H. (2009). Aging effect on the mobility and bioavailability of copper in soil. J. Environ. Sci..

[75] Ayangbenro A.S., Babalola O.O. (2017). A New Strategy for Heavy Metal Polluted Environments: A Review of Microbial Biosorbents. Int. J. Environ. Res. Public Health.

[76] Mishra J., Singh R., Arora N.K. (2017). Alleviation of Heavy Metal Stress in Plants and Remediation of Soil by Rhizosphere Microorganisms. Front. Microbiol..

[77] Moreira H., Pereira S.I., Marques A.P., Rangel A.O., Castro P.M. (2018). Effects of soil sterilization and metal spiking in plant growth promoting rhizobacteria selection for phytotechnology purposes. Geoderma.

[78] Brunetti G., Farrag K., Rovira P.S., Nigro F., Senesi N. (2011). Greenhouse and field studies on Cr, Cu, Pb and Zn phytoextraction by *Brassica napus* from contaminated soils in the Apulia region, Southern Italy. Geoderma.

[79] Turan M., Esringü A. (2008). Phytoremediation based on canola (*Brassica napus* L.) and Indian mustard (*Brassica juncea* L.) planted on spiked soil by aliquot amount of Cd, Cu, Pb, and Zn. Plant Soil Environ..

[80] Novo L.A.B., Covelo E.F., González L. (2013). Phytoremediation of amended copper mine tailings with *Brassica juncea*. Int. J. Mining Reclam. Environ..

[81] Li Z., Cao H., Yuan Y., Jiang H., Hu Y., He J., Zhang Y., Tu S. (2021). Combined passivators regulate the heavy metal accumulation and antioxidant response of Brassica chinensis grown in multi-metal contaminated soils. Environ. Sci. Pollut. Res..

[82] Baran A., Tarnawski M. (2015). Assessment of heavy metals mobility and toxicity in contaminated sediments by sequential extrac-tion and a battery of bioassays. Ecotoxicology.

[83] Ye S., Zeng G., Wu H., Zhang C., Dai J., Liang J., Yu J., Ren X., Yi H., Cheng M. (2017). Biological technologies for the remediation of co-contaminated soil. Crit. Rev. Biotechnol..

[84] Li L., Zhang K., Gill R.A., Islam F., Farooq M.A., Wang J., Zhou W. (2018). Ecotoxicological and Interactive Effects of Copper and Chromium on Physiochemical, Ultrastructural, and Molecular Profiling in *Brassica napus* L. BioMed Res. Int..

[85] Üçüncü A., Özkan A., Ölmez T., Tunca E., Gupta D.K., Chatterjee S. (2014). Phytoremediation of multiply metal-contaminated environments: Synergistic and competitive effects between heavy metals during uptake and transport. Heavy Metal Remediation.

[86] Marchiol L., Sacco P., Assolari S., Zerbi G. (2004). Reclamation of Polluted Soil: Phytoremediation Potential of Crop-Related *Brassica* Species. Water Air Soil Pollut..

[87] Bassegio C., Campagnolo A., Schwantes D., Junior A., Manfrin J., da Paz Schiller A., Bassegio D. (2019). Growth and accumulation of Pb by roots and shoots of *Brassica juncea* L. Int. J. Phytoremed..

[88] Belouchrani A.S., Mameri N., Abdi N., Grib H., Lounici H., Drouiche N. (2016). Phytoremediation of soil contaminated with Zn using Canola (*Brassica napus* L). Ecol. Eng..

[89] Diarra I., Kotra K.K., Prasad S. (2021). Assessment of biodegradable chelating agents in the phytoextraction of heavy metals from multi–metal contaminated soil. Chemosphere.

[90] Saraswat S., Rai J.P. (2009). Phytoextraction potential of six plant species grown in multimetal contaminated soil. Chem. Ecol..

[91] Gisbert C., Clemente R., Navarro-Aviñó J., Baixauli C., Ginér A., Serrano R., Walker D., Bernal M.P. (2006). Tolerance and accumulation of heavy metals by Brassicaceae species grown in contaminated soils from Mediterranean regions of Spain. Environ. Exp. Bot..

[92] Podar D., Ramsey M.H., Hutchings M.J. (2004). Effect of cadmium, zinc and substrate heterogeneity on yield, shoot metal concentration and metal uptake by *Brassica juncea*: Implications for human health risk assessment and phytoremediation. New Phytol..

[93] Bhattacharyya P.N., Jha D.K. (2011). Plant growth-promoting rhizobacteria (PGPR): Emergence in agriculture. World J. Microbiol. Biotechnol..

[94] Rajkumar M., Prasad M.N.V., Sandhya S., Freitas H. (2013). Climate change driven plant-metal-microbe interactions. Environ. Int..

[95] Hrynkiewicz K., Baum C., Malik A., Grohmann E. (2011). The potential of rhizosphere microorganisms to promote the plant growth in disturbed soils. Environmental Protection Strategies for Sustainable Development, Strategies for Sustainability.

[96] Lodewyckx C., Vangronsveld J., Porteous F., Moore E.R.B., Taghavi S., Mezgeay M., van der Lelie D. (2002). Endophytic bacteria and their potential applications. Crit. Rev. Plant Sci..

[97] Mendoza-Hernández J.C., Vázquez-Delgado O.R., Castillo-Morales M., Varela-Caselis J.L., Santamaría-Juárez J.D., Olivares-Xometl O., Morales J.A., Pérez-Osorio G. (2019). Phytoremediation of mine tailings by *Brassica juncea* inoculated with plant growth-promoting bacteria. Microbiol. Res..

[98] Franchi E., Cosmina P., Pedron F., Rosellini I., Barbafieri M., Petruzzelli G., Vocciante M. (2018). Improved arsenic phytoextraction by combined use of mobilizing chemicals and autochthonous soil bacteria. Sci. Total. Environ..

[99] Yu S., Teng C., Bai X., Liang J., Song T., Dong L., Jin Y., Qu J. (2017). Optimization of siderophore production by *Bacillus* sp. PZ-1 and its potential enhancement of phytoextration of Pb from soil. J. Microbiol. Biotechn..

[100] Dąbrowska G., Hrynkiewicz K., Trejgell A., Baum C. (2017). The effect of plant growth-promoting rhizobacteria on the phytoextraction of Cd and Zn by *Brassica napus* L. Int. J. Phytoremediat..

[101] Jinal H.N., Gopi K., Prittesh P., Kartik V.P., Amaresan N. (2019). Phytoextraction of iron from contaminated soils by inoculation of iron-tolerant plant growth-promoting bacteria in *Brassica juncea* L. Czern. Environ. Sci. Pollut. Res..

[102] Tang L., Hamid Y., Zehra A., Shohag J.I., He Z., Yang X. (2020). Endophytic inoculation coupled with soil amendment and foliar inhibitor ensure phytoremediation and argo-production in cadmium contaminated soil under oilseed rape-rice rotation system. Sci. Total. Environ..

[103] Shi Y., Xie H., Cao L., Zhang R., Xu Z., Wang Z., Deng Z. (2016). Effects of Cd- and Pb-resistant endophytic fungi on growth and phytoextraction of Brassica napus in metal-contaminated soils. Environ. Sci. Pollut. Res..

[104] Konkolewska A., Piechalak A., Ciszewska L., Antos-Krzemińska N., Skrzypczak T., Hanć A., Sitko K., Małkowski E., Barałkiewicz D., Małecka A. (2020). Combined use of companion planting and PGPR for the assisted phytoextraction of trace metals (Zn, Pb, Cd). Environ. Sci. Pollut. Res..

[105] Belimov A.A., Shaposhnikov A.I., Azarova T.S., Makarova N.M., Safronova V.I., Litvinskiy V.A., Nosikov V.V., Zavalin A.A., Tikhonovich I.A. (2020). Microbial consortium of PGPR, rhizobia and arbuscular mycorrhizal fungus makes pea mutant SGECdt comparable with Indian Mustard in cadmium tolerance and accumulation. Plants.

[106] Liu Y., Gao J., Bai Z., Wu S., Li X., Wang N., Du X., Fan H., Zhuang G., Bohu T. (2021). Unraveling Mechanisms and Impact of Microbial Recruitment on Oilseed Rape (*Brassica napus* L.) and the Rhizosphere Mediated by Plant Growth-Promoting Rhizobacteria. Microorganisms.

[107] Alberton D., Valdameri G., Rotuno Moure V., Monteiro R.A., de Oliveira Pedrosa F., Müller-Santos M., de Souza E.M. (2020). What Did We Learn from Plant Growth-Promoting Rhizobacteria (PGPR)-Grass Associations Studies Through Proteomic and Metabolomic Approaches?. Front. Sustain. Food Syst..

[108] Wu L.H., Lou Y.M., Xing X.R., Christie P. (2004). EDTA-enhanced phytoremediation of heavy metal contaminated soil with Indian mustrard and associated potential leaching risk. Agric. Ecosyst. Environ..

[109] Means J.L., Kucak T., Crerar D.A. (1980). Relative degradation rates of NTA, EDTA and DTPA and environmental applications. Environ. Pollut. Ser. B.

[110] Brynhildsen L., Rosswall T. (1997). Effects of metals on the microbial mineralization of organic acids. Water Air Soil Pollut..

[111] Zeremski-Škorić T., Sekulić P., Maksimović I., Šeremešić S., Ninkov J., Milić S., Vasin J. (2010). Chelate-assisted phytoextraction: Effect of EDTA and EDDS on copper uptake by *Brassica napus* L. J. Serbian Chem. Soc..

[112] Guo D., Ali A., Ren C., Du J., Li R., Lahori A.H., Xiao R., Zhang Z., Zhang Z. (2019). EDTA and organic acids assisted phytoextraction of Cd and Zn from a smelter contaminated soil by potherb mustard (*Brassica juncea* Coss.) and evaluation of its bioindicators. Ecotoxicol. Environ. Safe..

[113] Guarino C., Sciarrillo R. (2017). The effectiveness and efficiency of phytoremediation of a multicontaminated industrial site: Porto Marghera (Venice Lagoon, Italy). Chemosphere.

[114] Mishra R., Datta S.P., Annapurna K., Meena M.C., Dwivedi B.S., Golui D., Bandyopadhyay K. (2019). Enhancing the effectiveness of zinc, cadmium, and lead phytoextraction in polluted soils by using amendments and microorganisms. Environ. Sci. Pollut. Res..

[115] Bouquet D., Braud A., Lebeau T. (2017). Brassica juncea tested on urban soils moderately contaminated by lead: Origin of contamination and effect of chelates. Int. J. Phytoremediat..

[116] Chatuverdi R., Favas P., Pratas J., Varun M., Paul M.S. (2019). EDTA-assisted metal uptake in *Raphanus sativus* L. and *Brassica oleracea* L.: Assessment of toxicity and food safety. Bull. Environ. Contam. Toxicol..

[117] Niinae M., Nishigaki K., Aoki K. (2008). Removal of Lead from Contaminated Soils with Chelating Agents. Mater. Trans..

[118] Bucheli-Witschel M., Egli T. (2021). Environmental fate and microbial degradation of aminopolycarboxylic acids. FEMS Microbiol. Rev..

[119] Rathore S.S., Shekhawat K., Dass A., Kandpal B.K., Singh V.K. (2017). Phytoremediation Mechanism in Indian Mustard (*Brassica juncea*) and Its Enhancement Through Agronomic Interventions. Proc. Natl. Acad. Sci. India Sect. B Boil. Sci..

[120] Zeng X., Zou D., Wang A., Zhou Y., Liu Y., Li Z., Liu F., Wang H., Zeng Q., Xiao Z. (2020). Remediation of cadmium-contaminated soils using *Brassica napus*: Effect of nitrogen fertilizers. J. Environ. Manag..

[121] Chen S., Xu M., Ma Y., Yang J. (2007). Evaluation of different phosphate amendments on availability of metals in contaminated soil. Ecotoxicol. Environ. Saf..

[122] Valipour M., Shahbazi K., Khanmirzaei A. (2016). Chemical immobilization of lead, cadmium, copper, and nickel in contaminated soils by phosphate amendments. CLEAN–Soil Air Water.

[123] Niazi N.K., Bibi I., Fatimah A., Shahid M., Javed M.T., Wang H., Ok Y.S., Bashir S., Murtaza B., Saqib Z. (2017). Phosphate-assisted phytoremediation of arsenic by *Brassica napus* and *Brassica juncea*: Morphological and physiological response. Int. J. Phytoremediat..

[124] Cárdenas-Aguiar E., Suárez G., Paz-Ferreiro J., Askeland M., Méndez A., Gascó G. (2020). Remediation of mining soils by combining Brassica napus growth and amendment with chars from manure waste. Chemosphere.

[125] Gascó G., Álvarez M., Paz-Ferreiro J., Méndez A. (2019). Combining phytoextraction by *Brassica napus* and biochar amendment for the remediation of a mining soil in Riotinto (Spain). Chemosphere.

[126] Brunetti G., Farrag K., Soler-Rovira P., Ferrara M., Nigro F., Senesi N. (2012). The effect of compost and Bacillus licheniformis on the phytoextraction of Cr, Cu, Pb and Zn by three Brassicaceae species from contaminated soils in the Apulia region, Southern Italy. Geoderma.

[127] Van Der Ent A., Baker A.J.M., Reeves R.D., Pollard A.J., Schat H. (2012). Hyperaccumulators of metal and metalloid trace elements: Facts and fiction. Plant Soil.

[128] Bian F., Zhong Z., Zhang X., Yang C. (2017). Phytoremediation potential of moso bamboo (*Phyllostachys pubescens*) intercropped with *Sedum plumbizincicola* in metal-contaminated soil. Environ. Sci. Pollut. Res..

[129] Zhang N.N., Sun Y.M., Li L., Wang E.T., Chen W.X., Yuan H.L. (2010). Effects of intercropping and Rhizobium inoculation on yield and rhizosphere bacterial community of faba bean (*Vicia faba* L.). Biol. Fertil. Soils.

[130] Cao X., Luo J., Wang X., Chen Z., Liu G., Khan M.B., Kang K.J., Feng Y., He Z., Yang X. (2020). Responses of soil bacterial community and Cd phytoextraction to a *Sedum alfredii*-oilseed rape (*Brassica napus* L. and *Brassica juncea* L.) intercropping system. Sci. Total. Environ..

[131] Vannucchi F., Francini A., Raffaelli A., Sebastiani L. (2021). Removal of multi-contaminants from water by association of poplar and *Brassica* plants in a short-term growth chamber experiment. Environ. Sci. Pollut. Res..

[132] Quartacci M.F., Micaelli F., Sgherri C. (2014). *Brassica carinata* planting pattern influences phytoextraction of metals from a multi-ple contaminated soil. Agrochimica.

[133] Martínez-Alcalá I., Clemente R., Bernal M.P. (2020). Interactions between the Hyperaccumulator *Noccaea caerulescens* and *Brassica juncea* or *Lupinus albus* for Phytoextraction. Agronomy.

[134] Marques A., Caetano N., Castro P., Hussain C.M. (2020). Strategies for Enhancing Soil Phytoremediation and Biomass Valorization. The Handbook of Environmental Remediation—Classic and Modern Techniques.

[135] Song U., Park H. (2017). Importance of biomass management acts and policies after phytoremediation. J. Ecol. Environ..

[136] Kovacs H., Szemmelveisz K. (2017). Disposal options for polluted plants grown on heavy metal contaminated brownfield lands—A review. Chemosphere.

[137] Chatterjee N., Flury M., Hinman C., Cogger C.G. (2013). Chemical and Physical Characteristics of Compost Leachates, A Review Report Prepared for the Washington State Department of Transportation, Washington State University, USA. https://wsdot.wa.gov/Research/Reports/800/819.1.htm.

[138] Krueger E., Darland J., Goldyn S., Swanson R., Lehmann R., Shepardson S., Karpovich D. (2013). Water Leaching of Chelated Pb Complexes from Post-Phytoremediation Biomass. Water Air Soil Pollut..

[139] Vocciante M., Caretta A., Bua L., Bagatin R., Franchi E., Petruzzelli G., Ferro S. (2019). Enhancements in phytoremediation tech-nology: Environmental assessment including different options of biomass disposal and comparison with a consolidated approach. J. Environ. Manag..

[140] Liu Z., Wang L., Xiao H., Guo X., Urbanovich O., Nagorskaya L., Li X. (2020). A review on control factors of pyrolysis technol-ogy for plants containing heavy metals. Ecotoxicol. Environ. Safe..

[141] Dilks R., Monette F., Glaus M. (2016). The major parameters on biomass pyrolysis for hyperaccumulative plants—A review. Chemosphere.

[142] Yadav K.K., Gupta N., Kumar A., Reece L., Singh N., Rezania S., Khan S. (2018). Mechanistic understanding and holistic approach of phytoremediation: A review on application and future prospects. Ecol. Eng..

[143] Huang H., Yao W., Li R., Ali A., Du J., Guo D., Xiao R., Guo Z., Zhang Z., Awasthi M.K. (2018). Effect of pyrolysis temperature on chemical form, behavior and environmental risk of Zn, Pb and Cd in biochar produced from phytoremediation residue. Bioresour. Technol..

[144] Özkan A., Günkaya Z., Banar M. (2016). Pyrolysis of Plants After Phytoremediation of Contaminated Soil with Lead, Cadmium and Zinc. Bull. Environ. Contam. Toxicol..

[145] Özkan A., Çokaygil Z., Banar M. (2015). Stabilization of metal processing plant sludge via sequential application of phytoremediation and pyrolysis. Toxicol. Environ. Chem..

[146] Zhang Y., Chen Z., Xu W., Liao Q., Zhang H., Hao S., Chen S. (2020). Pyrolysis of various phytoremediation residues for biochars: Chemical forms and environmental risk of Cd in biochar. Bioresour. Technol..

[147] Nzihou A., Stanmore B. (2013). The fate of heavy metals during combustion and gasification of contaminated biomass—A brief review. J. Hazard. Mater..

[148] Willscher S., Mirgorodsky D., Jablonski L., Ollivier D., Merten D., Büchel G., Wittig J., Werner P. (2013). Field scale phytoremediation experiments on a heavy metal and uranium contaminated site, and further utilization of the plant residues. Hydrometallurgy.

[149] Spliethoff H., Scheurer W., Hein K. (2000). Effect of Co-Combustion of Sewage Sludge and Biomass on Emissions and Heavy Metals Behaviour. Process. Saf. Environ. Prot..

[150] Xu Y., Yang K., Zhou J., Zhao G. (2020). Coal-Biomass Co-Firing Power Generation Technology: Current Status, Challenges and Policy Implications. Sustainability.

[151] Edgar V.-N., Fabián F.-L., Mario P.-C., Ileana V.-R. (2021). Coupling Plant Biomass Derived from Phytoremediation of Potential Toxic-Metal-Polluted Soils to Bioenergy Production and High-Value by-Products—A Review. Appl. Sci..

[152] Dhiman S., Selvaraj C., Li J., Singh R., Zhao X., Kim D., Kim J.Y., Kang Y.C., Lee J.-K. (2016). Phytoremediation of metal-contaminated soils by the hyperaccumulator canola (*Brassica napus* L.) and the use of its biomass for ethanol production. Fuel.

[153] Encinar J.M., Pardal A., Sanchez N., Nogales S. (2018). Biodiesel by Transesterification of Rapeseed Oil Using Ultrasound: A Kinetic Study of Base-Catalysed Reactions. Energies.

[154] Wen D., Jiang H., Zhang K. (2009). Supercritical fluids technology for clean biofuel production. Prog. Nat. Sci..

[155] Angelova V., Ivanova R., Ivanov K. (2004). Heavy Metal Accumulation and Distribution in Oil Crops. Commun. Soil Sci. Plant Anal..

[156] Saka S., Kusdiana D. (2001). Biodiesel fuel from rapeseed oil as prepared in supercritical methanol. Fuel.

[157] Wang S., Wang J., Li J., Hou Y., Shi L., Lian C., Shen Z., Chen Y. (2021). Evaluation of biogas production potential of trace ele-ment-contaminated plants via anaerobic digestion. Ecotoxicol. Environ. Saf..

[158] Cao Z., Wang S., Wang T., Chang Z., Shen Z., Chen Y. (2014). Using Contaminated Plants Involved in Phytoremediation for Anaerobic Digestion. Int. J. Phytoremediat..

[159] Fiorentino G., Ripa M., Mellino S., Fahd S., Ulgiati S. (2014). Life cycle assessment of Brassica carinata biomass conversion to bio-energy and platform chemicals. J. Clean. Prod..

[160] Włóka D., Smol M., Kacprzak M. (2019). Energy efficiency of the phytoremediation process supported with the use of energy crops—*P. arundinacea* L. and Brassica napus L. Energy Policy.

[161] Cao X., Wang X., Tong W., Gurajala H.K., He Z., Yang X. (2019). Accumulation and distribution of cadmium and lead in 28 oilseed rape cultivars grown in a contaminated field. Environ. Sci. Pollut. Res..

[162] Glick B.R. (2014). Bacteria with ACC deaminase can promote plant growth and help to feed the world. Microbiol. Res..

[163] Wang L., Hou D., Shen Z., Zhu J., Jia X., Ok Y.S., Tack F.M.G., Rinklebe J. (2020). Field trials of phytomining and phytoremediation: A critical review of influencing factors and effects of additives. Crit. Rev. Environ. Sci. Technol..

